# Taguchi-based experimental investigation into weld cladding of Ni-WC MMC overlays by CMT process

**DOI:** 10.1007/s00170-022-09816-7

**Published:** 2022-09-03

**Authors:** Mohammad Reza Karimi, Sheng-Hui Wang, Jasmin Jelovica

**Affiliations:** 1 Department of Mechanical Engineering, The University of British Columbia, 6250 Applied Science Lane, Vancouver, BC V6T 1Z4 Canada; 2 Energy, Mining and Environment Research Centre, National Research Council of Canada, 4250 Wesbrook Mall, Vancouver, BC V6T 1W5 Canada; 3 Department of Civil Engineering, The University of British Columbia, 6250 Applied Science Lane, Vancouver, BC V6T 1Z4 Canada

**Keywords:** Weld cladding, Cold metal transfer (CMT), Carbide transfer efficiency, Retained WC, Carbide dissolution, Taguchi design of experiments

## Abstract

In the search for versatile and effective weld cladding processes to deposit ultra-wear-resistant Ni-WC MMC (Ni-based tungsten carbide metal matrix composite) overlays for mining applications, there is an increasing interest in exploring advanced low-heat-input cold metal transfer (CMT) method. Depositions of single weld bead tracks of Ni-WC MMCs on steel plates were performed by employing the CMT process; Taguchi’s design of experiments was used to plan the experimental investigation. All weld tracks exhibit continuous and uniform bead profile and sound metallurgical bonding to the substrate. Retained WCs are present in the overlay tracks relatively uniformly. The formation of primary WC and secondary carbides is observed depending on the level of dilution. In contrast to standard gas metal arc welding processes, the volume fraction of retained WC, which is negatively correlated with dilution level, is not directly interrelated with heat input for the CMT process and can reach a high level together with improved weld bead appearance at high deposition rate. Deposition rate has a positive correlation with average instantaneous power, which is, in turn, positively correlated with wire feed speed. The addition of oxygen into shielding gas mixtures promotes carbide transfer from cored feed wire to the weld track and increases the volume fraction of retained WC. Analysis of signal-to-noise ratios shows that it is difficult to find a single set of optimized processing parameters, and trade-offs are needed in engineering practice. The present investigation demonstrates that the Taguchi method is a powerful tool in process improvement for weld cladding of Ni-WC MMC overlays.

## Introduction

For hard-rock and oil-sands mining operations, machinery and structural components are often subjected to extremely harsh operational conditions and experience significant material losses, leading to increased equipment downtime [1]. For instance, damage caused by the wear of equipment and machinery in mining (including mining of oil-sands) and gas sectors in Canada was approximately C$2.5 billion/year in the 1980s [2]. In 2003, it was reported that this amount was over C$450 million for repair and maintenance of equipment at the main oil-sand-mining operators [3]. Thick protective overlays are increasingly used to protect machinery and equipment from damage under these harsh conditions. These typically come in the form of chromium carbide-based overlays (CCOs) and nickel-based metal matrix composites reinforced with WC (Ni-WC MMCs). These overlays can extend the service life of production-critical components, reducing costs related to maintenance, repair, and operations [4]. The material system of Ni-WC MMC is fundamentally different from CCOs in the way that WC is not formed in situ (through nucleation and growth) during the solidification process; instead, WC particles are incorporated in the feed materials and should remain unmelted during welding deposition [5]. Moreover, Ni-WC MMCs have higher wear resistance in comparison to CCOs and are used in applications where components work in more severe corrosive and erosive environments. The superior wear resistance of Ni-WC MMC overlays lies mainly in the existence of the retained hard WC particles in the overlays [6]. However, preferential dissolution of the WC particles incorporated in the feed material has been reported during the deposition of Ni-WC MMCs [7, 8]. The excessive dissolution of these particles significantly reduces the amount of retained hard WC carbides in the overlays and can lead to the formation of smaller and brittle W-rich carbides in the matrix during solidification [9], which adversely affects the wear resistance of the overlay [5]. In general, the degree of WC dissolution is inversely proportional to the level of heat input (*HI*) [10, 11]; therefore, deposition techniques featuring lower *HI* are sought for Ni-WC MMCs.

Welding-based processes that are employed to deposit Ni-WC MMC are laser cladding [12], plasma transferred arc welding (PTAW) [13], gas tungsten arc welding (GTAW) [14], and gas metal arc welding (GMAW) [15]. WC dissolution is reported for all of these processes. Laser cladding and PTAW processes could potentially deposit high-quality Ni-WC MMC overlays due to their low *HI*; however, some related limitations make their applications for onsite repairs problematic or implausible. For instance, powder-based PTAW and laser cladding processes may only be utilized for the cases of flat welding positions; besides, they are susceptible to wind dispersion and not suited for large-size components, and have high capital and operating costs [16]. On the other hand, GMAW as a wire electrode-based process is a versatile and comparatively cheaper method, applicable for all welding positions, has a high deposition rate (*DR*) and requiring smaller-size power supplies. Nevertheless, high WC dissolution is reported for the GMAW process [5] due to the high *HI*.

For the GMAW cladding process, the relationship between WC dissolution and welding parameters (including *HI*) and other related factors (such as protection gases and substrate chemistry) is complicated. Choi et al. [4] achieved a high WC volume fraction while employing minimum *HI* to deposit Ni-WC MMC overlays by GMAW process, which led to less than 1% dilution, insufficient for oil-sand mining where high impact loading is often present. Badisch and Kirchgaßner [17] and Choi et al. [4] reported that dissolution of WC occurs during the GMAW process, regardless of whether the overlay was deposited over a steel plate or previously deposited Ni-WC MMC overlays. Vespa et al. [18] and Badisch and Kirchgaßner [17] determined that increasing wire feed speed (*WFS*) and welding voltage results in an increasing dilution and decreasing carbide volume fraction in the Ni-WC MMC overlays. Günther and Bergmann [19] observed that welding travel speed (*TS*) has no significant effect on the dilution for the studied parameters ranges. WC dissolution can occur during the droplet transfer and within the molten weld pool. As a result, to achieve a high-quality Ni-WC MMC overlay, *HI* must be kept to a minimum to limit the WC dissolution while maximizing their transfer efficiency [20]. However, adjusting the welding parameters to achieve the minimum droplet temperature does not guarantee the mitigation of WC dissolution. Scott [21] utilized welding parameters that led to the lowest *HI* but still observed partial dissolution of WC with poor weld bead appearance despite improved volume fraction of retained WC (*f*_WC_). Guest [20] compared the effects of different shielding gas (*SG*) mixtures for depositing Ni-WC MMC and reported a significant increase in carbide transfer efficiency (*η*) for Ar + O_2_ in comparison to Ar + CO_2_, showing a beneficial effect on O_2_.

Besides the metallurgical dissolution of WC as discussed above, Guest [20, 22] also documented the non-wetting behavior of WC, resulting in a portion of WC particles not being caught up in the molten pool during the welding process, leading to low *f*_WC_ in Ni-WC MMC overlays due to mechanical loss of WC particles. This phenomenon was also reported by Günther et al. [23].

Weld cladding is a multi-factor, multi-response process. A substantial amount of time and cost is required to determine the optimal welding envelope by integrating different welding control parameters through trial and error [24]. Design of experiments (DOE) is one of the various methods to correlate input factors with responses [25]. Taguchi design is a widely accepted DOE method that utilizes orthogonal arrays to reduce the number of experiments.

Cold metal transfer (CMT) is a relatively new advancement of the GMAW process, enabling welding operations with reduced *HI*. CMT process is equipped with a highly precise waveform control system and a fast mechanically retracting wire feeding system that allows detachment of one droplet at a time and significantly reduces *HI*, distortion, and spatter [26]. CMT process has been utilized for various applications in industry, such as welding dissimilar materials [27], joining thin sheet metal [28], aluminum welding [29], and hardfacing [30]. With the CMT process, researchers achieved a low dilution level (*DL*) with a high *DR* for a set of experiments with low *HI* [31], mitigating the formation of the unwanted intermetallic layer in aluminum/steel welding [32]. It should be mentioned that the control of welding parameters of the CMT process involves a synergistic effect and is much more sophisticated compared to standard GMAW processes. According to Ola and Doern [33], while maintaining other parameters constant during depositing Ni-based INCONEL 718 superalloy overlay by the CMT process, *HI* is linearly related to *WFS*, which affects dilution and weld bead geometry; the contact angle (*θ*) was found to be significant in generating defect-free clad surfaces.

Considering the significance of *HI* in relation to WC dissolution, there is a strong interest in the deposition of Ni-WC MMC overlays by the CMT process. To the best of the authors’ knowledge, no articles have been published yet on depositing Ni-WC MMC overlays using tubular cored feed wire by this low-*HI* process. Therefore, it is necessary to perform experimental explorations to have a comprehensive understanding of the technological characteristics of weld cladding of resulting overlays by the CMT process: on the one hand, due to its uniqueness as compared to the standard GMAW processes, it is not clear how well the knowledge gained previously on welding cladding of Ni-WC MMC overlays by standard GMAW processes can be transferred to the case with CMT; on the other hand, due to the complex nature of weld cladding of related overlays as discussed above, it is crucial to find a suitable operating envelope to assure Ni-WC MMC overlay quality, such as minimizing the dissolution of the WC in the overlay without sacrificing other quality criteria if CMT process is engaged. While overall investigations are ongoing to compare CMT technology with standard GMAW in relation to the weld cladding processes, this paper reports processing development efforts to achieve high-quality Ni-WC MMC overlays by CMT process; single-bead Ni-WC MMC overlays were deposited for the investigation, and Taguchi design of experiments was used to systematically investigate the effect of welding parameters under CMT synergic mode on WC dissolution, *η*, *DL*, *DR*, and weld bead geometry.

## Methodology

### Materials and consumables

ASTM A36 hot rolled flat bar steel [34] is used as a substrate with the size of 100 mm × 75 mm × 10 mm. The surface of the substrate is ground, degreased, and cleaned with acetone prior to the welding. The feed material is a tubular Ni-based metal cored wire with a diameter of 1.6 mm and with WC particles incorporated in the core [35]. The chemical composition and specification of the substrate and the tubular cored feed wire are listed in Tables 1 and 2, respectively.

**Table 1:** Chemical composition of ASTM A36 steel [34]

[wt.%]	C	P	S	Si	Cu	Fe
ASTM A36	0.26	0.04	0.05	0.4	0.20	Bal

**Table 2:** Tubular cored wire specification [35]

Electrode commercial name	Supplier	Nominal composition	Nominal hardness
COR®FACE 164 MC	COR-MET	NiCrBSi-55 wt.% WC	Matrix = 38–42 HRC WC = 2600 Knoop

### Experimental setup and design

**Fig. 1:**
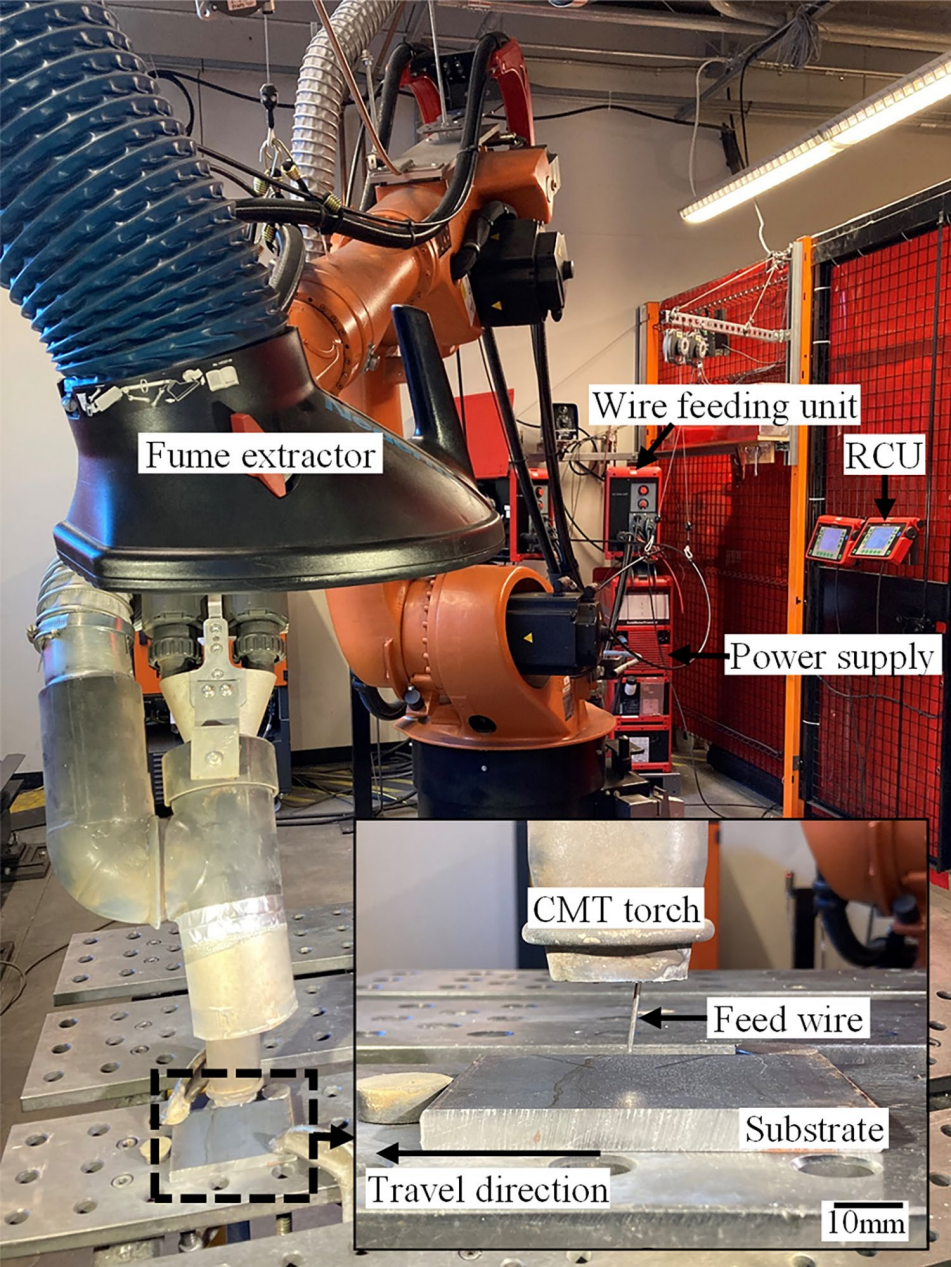
Snapshot of CMT welding machine and KUKA robot at NRC-Vancouver

As shown in Fig. 1, the welding system includes a Fronius TransPuls Synergic 5000 power supply with a RCU 5000i remote control unit and Fronius VR 7000 CMT wire feeding unit. The deposition process is carried out using a 6-axis KUKA robot KR 60 HA and KR C4 control system with a welding torch (Robacta Twin Compact Pro torch neck) mounted on its tool flange. This allows for fully automatic control of the welding process. Real-time welding current and arc voltage are measured at a sample rate of 20 kHz. The welding torch was positioned perpendicular to the substrate, which allowed a continuous supply of the feed wire during welding deposition while keeping the angle between the wire and the substrate at 82°. Contact tip to work distance was kept at 15 mm for all experiments.

**Fig. 2:**
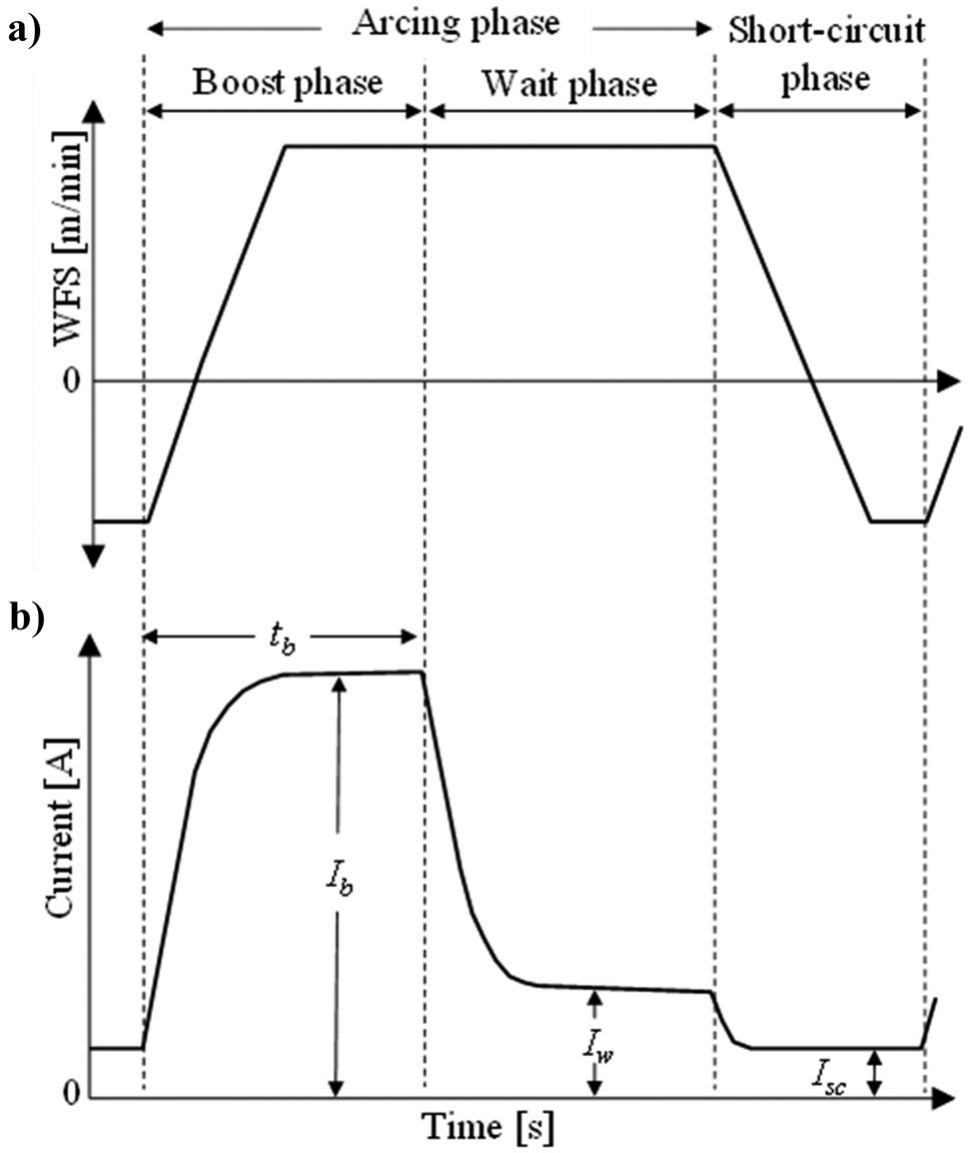
Typical CMT waveform cycle: **a** *WFS* vs time (negative value corresponding to electrode retracting); **b** current vs time (*I*_b_: boost current; *t*_w_: boost duration; *I*_w_: wait current; *I*_sc_: short-circuit current)

Based on the previous in-house knowledge of depositing Ni-WC MMC at the National Research Council of Canada (NRC)-Vancouver, the CMT synergic mode (CrNi19 12 cladding, ref. 1633) is set for conducting the experiments. The synergic mode is a pre-programmed operation mode. Once the electrode type/diameter, welding process mode, *WFS*, and *SG* mixture are selected, the welding power supply automatically adjusts the current and voltage. Moreover, WFS in this paper refers to a preset (nominal) value. As mechanically retracting of the feed wire occurs repetitively (as indicated in Fig. 2a), the actual *WFS* can vary from the preset *WFS* in the CMT process [36].

The typical CMT waveform cycle is illustrated in Fig. 2. Characteristics of the cycle are listed in Table 3. The CMT cycle is defined as the period required to form and transfer a molten droplet into the weld pool, and it consists of two fundamental phases: short-circuiting and arcing phase. Arcing phase includes boost and wait phase. At the beginning of the boost phase, the current rapidly rises to the preset value (as indicated in Fig. 2b), reigniting the arc between electrode and substrate, and leading to the formation of a droplet at the tip of the electrode. Then, the electrode advances toward the weld pool while the current decreases to avoid globular transfer of the molten droplet; the voltage changes synergistically with the current. Once the molten droplet touches the weld pool (beginning of the short-circuit phase), the current immediately drops near zero, and the electrode retracts, allowing the detachment and transfer of the molten droplet into the weld pool [37]. Duration of short-circuit phase is nonadjustable, once a CMT synergic mode is selected [36].

**Table 3:** Preset waveform values for chosen *WFS*

Symbol	Unit	Definition	Synergic *WFS* guideline [m/min]			
			**2.1**	**3.9**	**5.7**	**7.5**
***I***_**w**_	[A]	Current setpoint for wait phase, until the molten droplet touches the weld pool	35	60	82	110
***I***_**sc**_	[A]	Current setpoint in the short-circuit phase	35	40	73	110
***I***_**b**_	[A]	Current setpoint in the boost phase	225	290	307	340
***t***_**b**_	[ms]	Maximum duration of the boost phase	3	3.75	5.13	5.6

Taguchi DOE L16 orthogonal array is used for the systematic evaluation of the welding control parameters. The experiments are focused on the effect of the *WFS*, *TS*, and *SG* mixture (each at 4 levels) while keeping the other variables constant. The available range of *WFS* for the CMT synergic mode (CrNi19 12 cladding, ref. 1633) is 2.1–7.5 m/min. Accordingly, 2.1, 3.9, 5.7, and 7.5 m/min is selected as the four levels of WFS. It should be noted that a selection of *WFS* outside of the available synergic mode range necessitates creating a user-defined characteristic, which is beyond the scope of this study. The breakdown of the preset welding current waveform characteristics for each of the chosen *WFS* is listed in Table 3; the voltage will vary accordingly. As indicated in Fig. 2b and Table 3, the current level and its features differ significantly within one waveform cycle from one phase to another.

For standard GMAW, *TS* is one of the three factors in arc welding that affects the *HI*, along with current and voltage. *TS* range should ensure a defect-free deposition without sacrificing *DR*. In this paper, *TS* is set to 0.3–0.6 m/min with 0.1 increments. Four *SG* mixtures are selected for the study: 25% CO_2_, 15% CO_2_, 2% O_2_, and 5% O_2_ (Ar balanced). For *SG* mixtures, commercially available Praxair’s Ar–O_2_ StarGold blend, CO_2_, and Ar gas cylinder were used. Table 4 lists the welding control parameters and their levels used to deposit 16 different bead-on-plate experiments using the CMT process for a length of 50 mm. All experiments were successfully deposited with no discontinuity in the weld bead or any visual defect. In addition to Taguchi L16 experiments, two confirmation tests were conducted to validate optimum processing conditions predicted by the Taguchi design (see Sections 3.4 and 3.5 for details).

**Table 4:** Taguchi L16 factors and levels

Std. order	*WFS* (m/min)	*TS* (m/min)	*SG* (%, Ar balanced)
1	2.1	0.3	25% CO_2_
2	2.1	0.4	15% CO_2_
3	2.1	0.5	2% O_2_
4	2.1	0.6	5% O_2_
5	3.9	0.3	15% CO_2_
6	3.9	0.4	25% CO_2_
7	3.9	0.5	5% O_2_
8	3.9	0.6	2% O_2_
9	5.7	0.3	2% O_2_
10	5.7	0.4	5% O_2_
11	5.7	0.5	25% CO_2_
12	5.7	0.6	15% CO_2_
13	7.5	0.3	5% O_2_
14	7.5	0.4	2% O_2_
15	7.5	0.5	15% CO_2_
16	7.5	0.6	25% CO_2_

### Characterization of the overlays

One of the critical features governing all welding processes is *HI*, which is often associated with the mechanical properties of the resultant overlay [20]. *HI* is a relative measure of the energy transferred per unit length of a weld. For pulsed GMAW or CMT processes with complex waveforms, the traditional method of calculating *HI* (using average current and voltage) may not give the same result as using instantaneous values [38, 39]. Therefore, to accurately capture the changes in welding waveforms, the real-time current and voltage data were acquired at 20 kHz. Average instantaneous power (*AIP*) and *HI* are then calculated using Eqs. (1) and (2), respectively.

1 $${AIP}\; [\mathrm{W}]= \mathrm{{Total\; instantaneous \;power}/n=\:^{\sum_{0}^{t}({\it{I}}_{i}\times{\it{V}}_{i})}/n}$$

2 $${HI}\;[\frac{kJ}{mm}]=\:^{\sum^{t}_{0}(I_\mathrm{i}\times V_\mathrm{i})\times\Delta t}/(t\times TS)$$

where *I*_*i*_ is the welding current, *V*_*i*_ is welding voltage for each sample, *n* is the number of samples, $\Delta t$ is the sampling interval, and *t* is welding time. According to Joseph et al. [39], Eq. (1) is a simplified form of *AIP* calculation by using discretized voltage and current samples. It should be noted that the waveform characteristics of the voltage and current vary with changing WFS for a selected synergic mode, and therefore so does the *HI*. It is believed that process efficiency is higher for CMT as compared to standard GMAW [39–41]. As no agreed-upon efficiency factor is yet reported for the CMT process, for simplicity it is assumed that *HI* takes the value of the arc energy. As the efficiency factor should be similar for all samples, the assumption made here should not affect the overall discussions on the subject matter.

*DR* is defined as the actual weight of material deposited per unit of time. The plate was weighed three times with 0.001 g precision before and after the deposition of each single weld bead track, and the average values were used in Eq. (3) to calculate the *DR*.

3 $${DR} \left[\frac{\mathrm{kg}}{\mathrm{h}}\right]=\frac{\mathrm{Final\; weight}-\mathrm{initial\; weight}}{t}$$

To investigate the microstructure and quality of the overlays, resultant samples were cut from three locations (first quarter, middle, and the third quarter of the weld bead track), hot mounted, ground, and polished. To reveal the fusion lines, samples were etched by 2% Nital. The cross-section was first locally photographed, and then a high-resolution image of the entire cross-section of the weld bead track at × 200 magnification was constructed using the auto-stitch feature of the Keyence VHX-7000 series digital microscope. The resultant images were used to calculate the *DL*, *f*_WC_, weld bead geometry, and porosity. The microstructures of tubular cored wire and the single-bead Ni-WC MMC overlays were investigated using scanning electron microscopy (SEM, HITACHI S-3500 N). Energy-dispersive X-ray spectrometer (EDS) capable of quantitative analysis and X-ray mapping was used to determine the composition of various phases in the samples.

The initial carbide volume fraction (*f*_p_) of cored feed wire is considered the theoretical maximum of retained carbides that can be attained in the resultant overlays. This value is required to calculate *η* and monitor the carbide loss (due to WC dissolution or non-wetting behavior). *f*_p_ is a function of the linear mass of the electrode (*l*_m_), powder weight percentage (*P*_p_), density of powder (*ρ*_p_), and nickel sheath area (*A*_Ni-sheath_), which can be calculated using Eqs. (4)–(6) [42].

4 $${l}_{\mathrm{m}}=\frac{\mathrm{Weight \;of \;electrode}}{\mathrm{Length \;of\; electrode}}$$

5 $${P}_{\mathrm{p}}=\frac{\mathrm{Weight\; of\; powder\; inside\; electrode}}{\mathrm{Weight \;of \;electrode}}$$

6 $${f}_{\mathrm{p}}=\frac{\left({P}_\mathrm{p}\times {l}_{\mathrm{m}}\right)}{\left({P}_{\mathrm{p}}\times {l}_{\mathrm{m}}\right)+\left({A}_{\mathrm{Ni-sheath}}\times {\rho }_{\mathrm{p}}\right)}\times 100\%$$

**Fig. 3:**
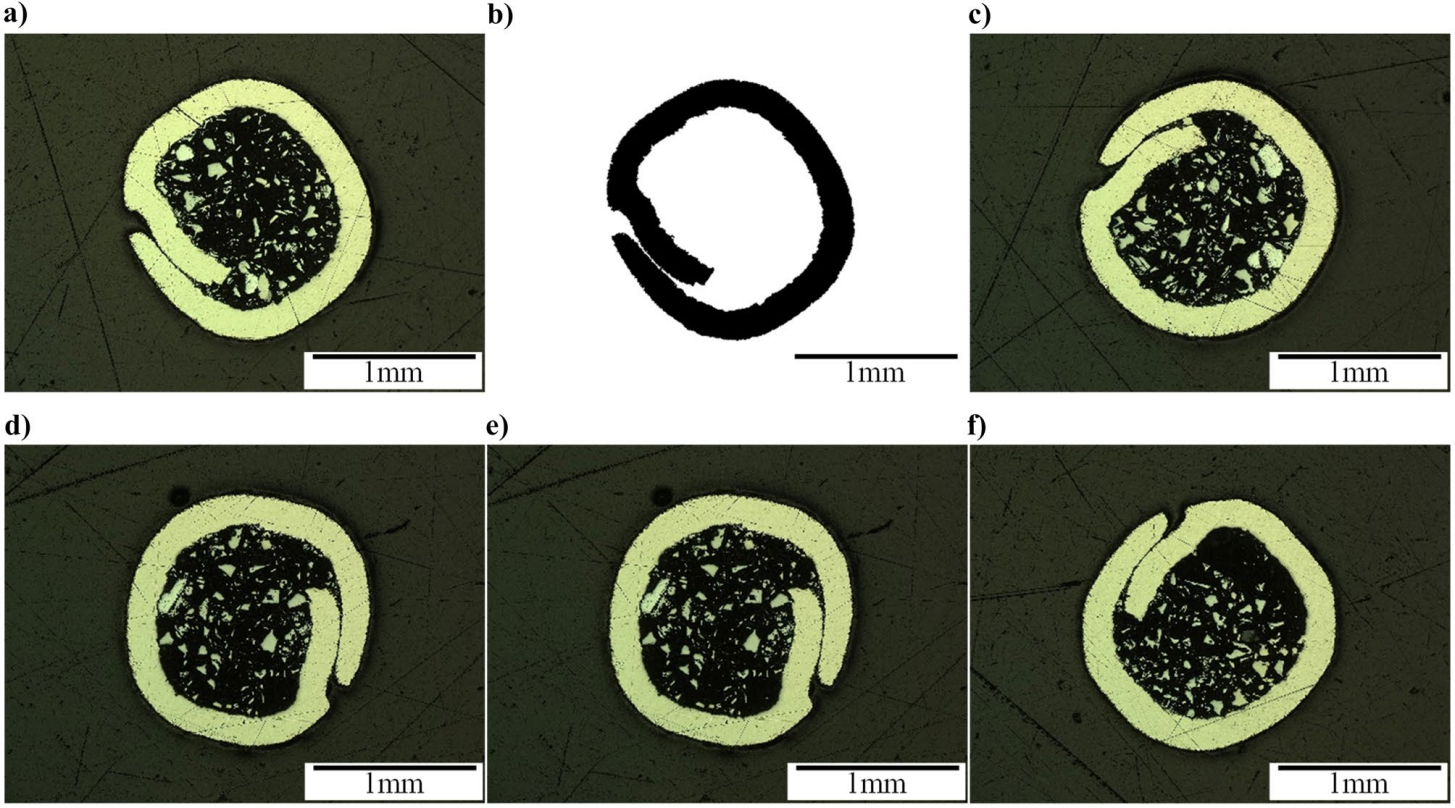
Optical microscope image of tubular Ni-WC cored wire cross-section; **a** original image of sample 1; **b** binary image of sample 1; **c–f** original images of samples 2–5

In order to find *f*_p_, five samples of about 100 mm in length were cut from the electrode and weighted with 0.001 g precision to find the weights of the electrode, Ni-sheath, and the powder. All measurements are repeated three times, and the average value is reported. *A*_Ni-sheath_ was measured by converting the cross-section image of the electrode (Fig. 3a) to the binary image (Fig. 3b) to facilitate measuring the Ni-sheath area in ImageJ software. Table 5 summarizes the measured values used to calculate the *f*_p_.

**Table 5:** Measured values of the tubular cored wire samples, used to calculate the *f*_p_

Measured parameters	Sample 1	Sample 2	Sample 3	Sample 4	Sample 5	Average
Sample length [mm]	96.54	102.12	106.70	97.52	101.43	100.86
Electrode weight [g]	1.631	1.739	1.821	1.658	1.705	1.711
Powder weight [g]	0.892	0.958	0.995	0.904	0.921	0.934
*l*_m_ [g/mm]	0.0169	0.0170	0.0171	0.0170	0.0168	0.0170
*A*_Ni-sheath_ [mm^2^]	0.792	0.808	0.835	0.830	0.830	0.819
*P*_p_ [%]	54.68	55.08	54.63	54.52	54.01	54.58

The analysis shows that *l*_m_ = 0.0170 g/mm, *P*_p_ = 54.58% (which is similar to manufacturer’s specification of 55 wt.% [35] as listed in Table 2), *ρ*_p_ = 16.67 g/cm^3^ (assuming that the powder consists of only WC) and *A*_Ni-sheath_ = 0.819 mm^2^. Using these values in Eqs. (4)–(6), *f*_*p*_ is 40.4%.

**Fig. 4:**
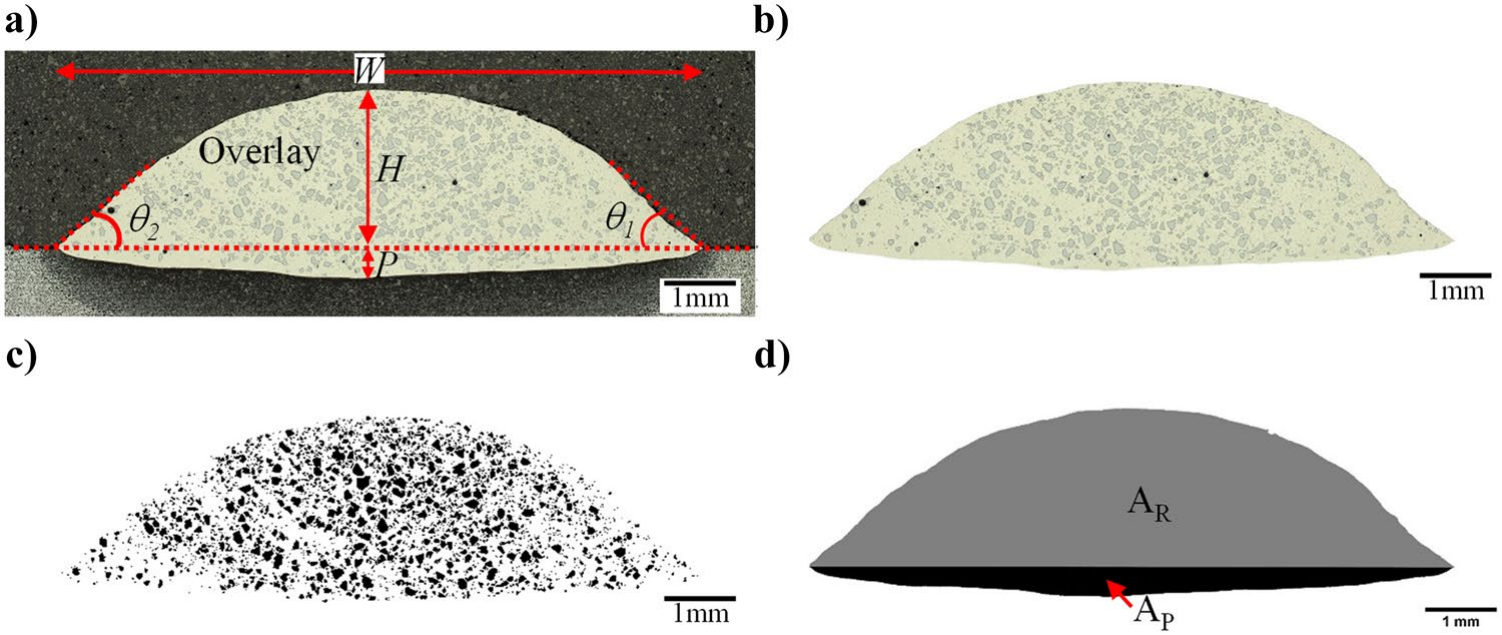
Typical overlay cross-section; **a** original image; **b** extracted overlay; **c** binary image of the carbides; **d** gray-scale dilution (*A*_R_ = reinforcement area; *A*_P_ = area of weld penetration; *W* = width; *H* = height; *P* = depth of penetration; *θ*_1_ and *θ*_2_ weld contact angle)

Weld bead geometry measurement is conducted over the three prepared sample cross-sections for each experiment, and the average value is reported. Figure 4a illustrates the width (*W*), height (*H*), penetration depth (*P*), and contact angle ($\theta =\frac{{\theta }_{1}+{\theta }_{2}}{2}$).

*f*_WC_ is calculated using Eq. (7). It is calculated as the ratio of *A*_WC_ (retained and primary WC area, see Fig. 4c) to the total overlay area (*A*_R_ + *A*_P_, see Fig. 4d). Furthermore, *η* is an important measure of the effectiveness of related weld cladding process. *η* is the fraction of WC in feed wire, which is transferred to the overlay. A small portion of carbides transferred to the overlay will be dissolved, forming primary and secondary carbides during the solidification process; in theory, retained, primary, and secondary WC should all be measured to determine *η*. However, for simplicity, $\eta$ is calculated using Eq. (8), by taking into account only retained and primary WC while ignoring the secondary WC (as the latter consists of only a small fraction of the total transferred carbides and is difficult to quantify). In other words, *η* measures the amount of retained and primary WC in the overlay relative to the maximum achievable value (i.e., *f*_p_).

7 $${f}_{\mathrm{WC}}\; (\%)=A_{\mathrm{WC}}/(A_{\mathrm{R}}+A_{\mathrm{P}})$$

8 $$\eta \;(\%)=\frac{A_{\mathrm{WC}}/A_{\mathrm{R}}}{f_{\mathrm{P}}}$$

ImageJ software was used to convert the optical images to binary images and to find the various areas of the carbide and the overlay (see Fig. 4c). As samples were lightly etched by 2% Nital, only retained and primary carbides can be easily identified by optical microscopy. They are not easily distinguishable, and both are captured during image processing. It should be noted that the amount of primary carbides is small compared to that of retained carbides.

Dilution is due to a small portion of base material being mixed with the deposited material. Based on cross-section characterization as illustrated in Fig. 4d, *DL* is calculated using Eq. (9). For this purpose, Adobe Photoshop was used to separate reinforcement and penetration areas, followed by ImageJ software to calculate these areas of the overlay cross-section. It should be mentioned that, while calculating $\eta$ using Eq. (8), the dilution effect is eliminated.

9 $$DL\;(\%)=A_{\mathrm{P}}/(A_{\mathrm{R}}+A_{\mathrm{P}})$$

### Taguchi's signal-to-noise ratio

In Taguchi design, the signal-to-noise (S/N) ratio reflects how a response (i.e., a quality criterion) fluctuates with a process control parameter (signal) under varying noise conditions (other factors). The goal of quality criteria can be described as follows: larger the better, nominal the best, and smaller the better [43]. In this paper, the larger the better is selected as the goal of experiments for *f*_WC_, *η*, *DR*, and *W*/*H*. For the *DL* and *P*, smaller the better is selected as the goal of the experiments. Accordingly, the S/N ratios for selected responses are calculated based on the formulas listed in Table 6. In general, higher S/N ratio levels indicate that a control parameter has a larger influence on the outcome (response) and/or that it minimizes the influence of noise components. Minitab 20.3 software was utilized to obtain the means of mean plots and means of S/N ratio plots.

**Table 6:** Summary of the selected goal for quality criteria of the overlay and corresponding S/N ratio formula

Quality criterion	Goal of experiments	S/N ratio^*^ formula
*f*_WC_ *η* *DR * *W*/*H*	Larger-the-better (maximize the response)	$\mathrm{S}/\mathrm{N}=-10\times \mathrm{log}(\Sigma (\frac{1}{{{\mathrm{Y}}_{\mathrm{i}}}^{2}})/\mathrm{n})$
*DL* *P*	Smaller-the-better (minimize the response)	$\mathrm{S}/\mathrm{N}=-10\times \mathrm{log}(\Sigma ({{\mathrm{Y}}_{\mathrm{i}}}^{2})/\mathrm{n})$

*n* = number of response's observations in each experiment

*Y*_i_ = observed measurement for each response

^*^ The unit of the S/N ratio is decibels-isotropic (dBi)

## Results and discussion

### Characterization of Ni-WC tubular cored feed wire

**Fig. 5:**
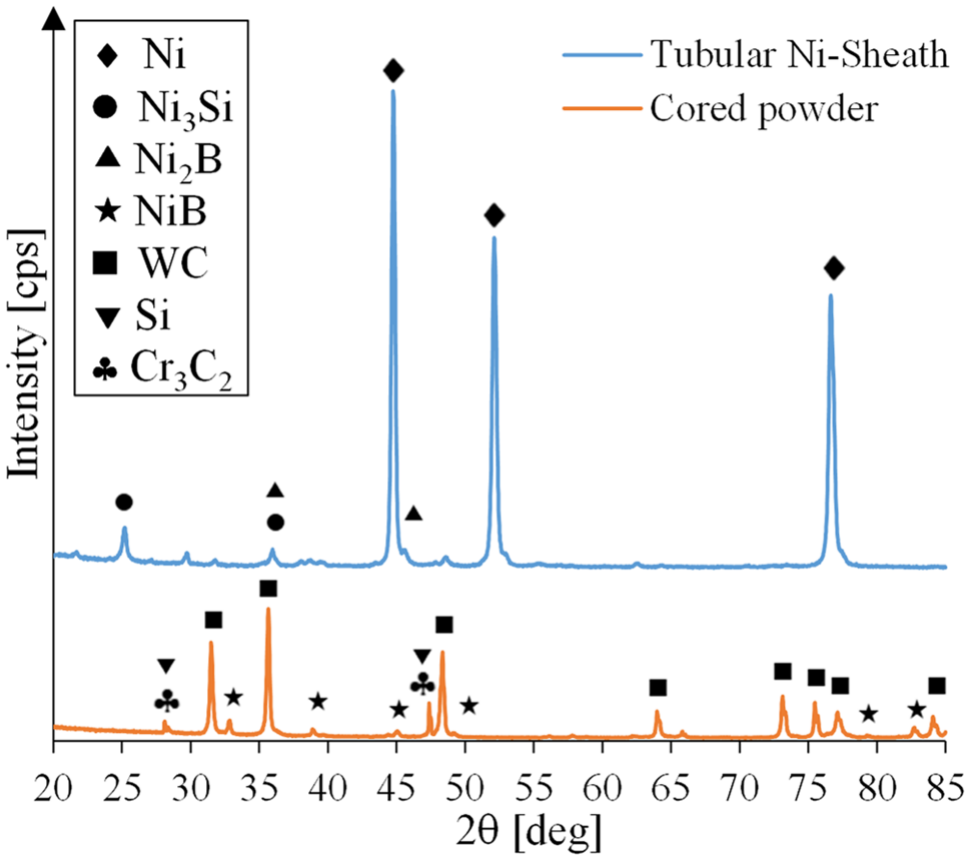
XRD patterns of tubular wire sheath and cored powders

**Fig. 6:**
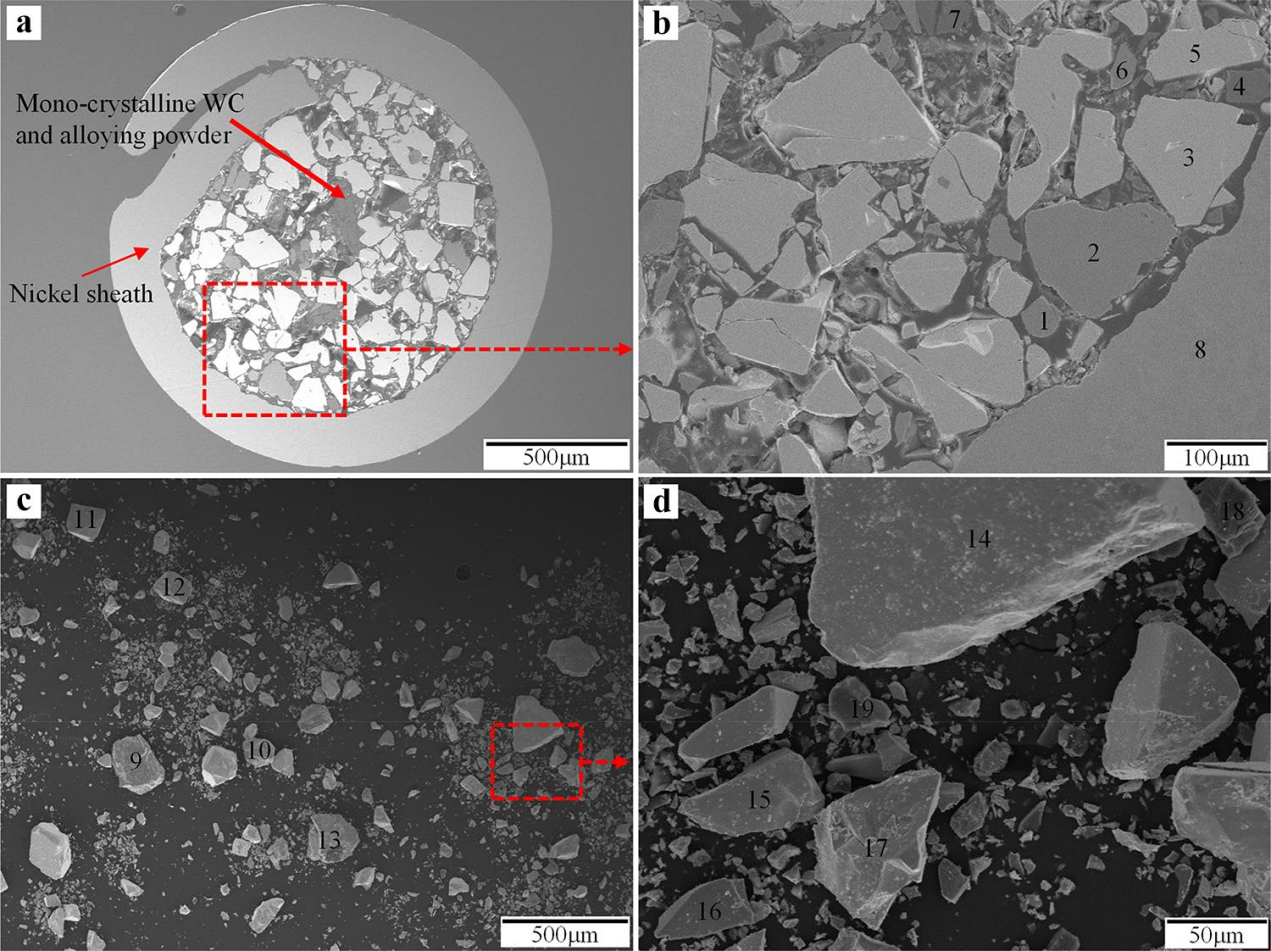
**a** SEM image of the cross-section of the tubular Ni-WC cored wire; **b** selected area in **a** at higher magnification; **c** powders taken out of the core of the feed wire; **d** selected area in **c** at higher magnification (with the numbers indicating the locations of EDS spot measurements)

X-ray diffraction (XRD) patterns of the sheath of the feed wire and cored powder are presented in Fig. 5, and the corresponding SEM micrographs are shown in Fig. 6. The wire sheath consists of Ni alloy with Ni_3_Si and Ni_2_B precipitates. Boron and silicon elements are usually added to reduce the melting point and improve the self-fluxing ability of the matrix [44]. Table 7 lists EDS spot measurement results of the core particles at the locations indicated in Fig. 6. The results show the presence of angular shape tungsten carbide (points 3, 5, 11, 12, 14, 15, and 17), Ni-rich (points 1, 6, 8, 9, 10, 13, and 16), Si-rich (points 4, 7, 18, and 19), and Cr-rich (point 2) particles. Based on XRD analysis, these particles are identified to be mono-crystalline tungsten carbide (WC), NiB, Si, and Cr_3_C_2_, respectively. In general, the particles/powders in the core of the feed wire consist of tungsten carbide particles and additional powders of alloying elements and/or fluxing agents. Mono-crystalline WC is normally fabricated by carburizing the conventional eutectoid W_2_C/WC particle (∼3.7 to 4.1 wt.% carbon) at elevated temperature to increase its carbon content to 6.1 wt.%. The mono-crystalline WC is more resistant to dissolution [10] and has better thermal stability [45] compared to eutectoid W_2_C/WC. However, they have lower hardness (1200–2100 HV) [5].

**Table 7:** EDS spot measurement results of marked locations in Fig. 6

Elements (wt.%)	Point																		
	**1**	**2**	**3**	**4**	**5**	**6**	**7**	**8**	**9**	**10**	**11**	**12**	**13**	**14**	**15**	**16**	**17**	**18**	**19**
**C**	6.16	3.68	9.11	8.98	9.55	5.87	10.56	5.57	9.44	11.23	12.91	11.49	7.84	9.05	9.22	5.41	11.39	11.84	14.5
**O**	0.12	2.34	0.55	0.39	0.76	0.05	0.77	0.71	0.00	1.41	1.02	1.49	2.04	1.09	0.88	0.31	1.82	1.33	0.95
**Si**	0.04	0.18	0.00	90.11	0.00	0.16	88.37	0.08	0.06	0.39	0.00	0.00	0.33	1.36	0.00	0.39	3.72	85.21	84.29
**Cr**	0.24	93.61	0.09	0.00	0.00	0.00	0.03	0.00	0.01	0.03	0.06	0.01	0.07	0.09	0.04	0.00	0.00	0.05	0.00
**Fe**	0.14	0.06	0.21	0.00	0.03	0.13	0.00	0.06	0.05	0.21	0.14	0.24	0.24	0.02	0.52	0.13	4.54	0.00	0.00
**Ni**	93.3	0.13	0.09	0.51	0.02	93.79	0.28	93.59	90.31	83.56	0.17	0.15	86.82	0.88	0.26	89.96	1.06	0.25	0.25
**W**	0.00	0.00	89.95	0.00	89.63	0.00	0.00	0.00	0.14	3.16	85.71	86.61	2.66	87.52	89.09	3.8	77.47	1.31	0.00

### Non-wetting behavior of powders during deposition

**Fig. 7:**
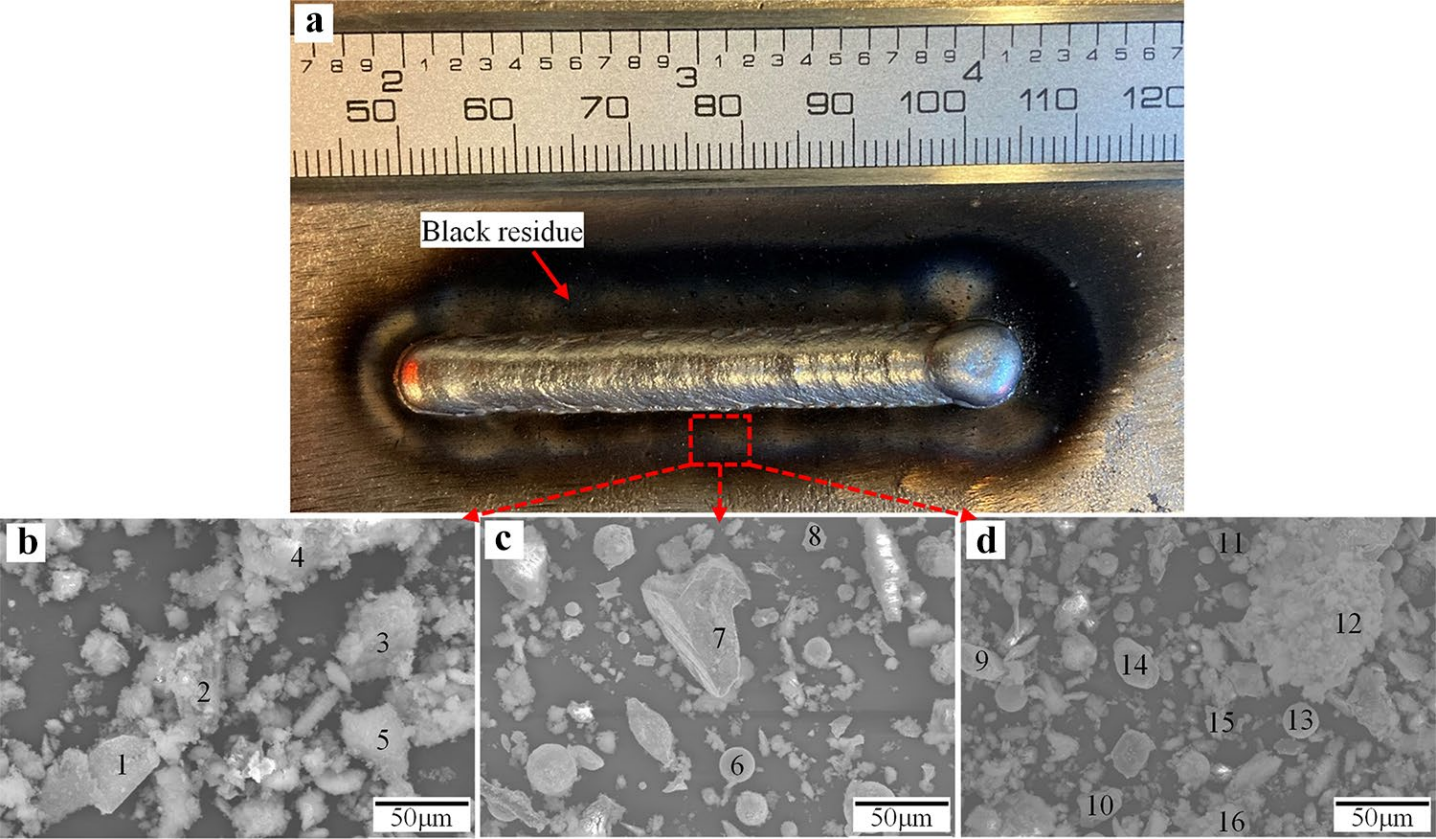
**a** Black residues around weld bead after deposition; **b–d** SEM images of black residues

A considerable amount of black residue is observed across the whole weld region for all samples. Figure 7a shows the top view of one of the bead-on-plate samples, including the black residue. Figure 7b–d show some SEM micrographs, and Table 8 lists EDS spot measurement results of some particles/locations (as indicated in Fig. 7b–d) of the residue.

**Table 8:** EDS spot measurement results of marked locations in Fig. 7

Elements (wt.%)	Point															
	**1**	**2**	**3**	**4**	**5**	**6**	**7**	**8**	**9**	**10**	**11**	**12**	**13**	**14**	**15**	**16**
**C**	7.58	15.88	7.34	8.52	10.38	6.07	9.97	12.37	16.23	16.28	2.33	9.51	18.1	5.87	7.63	7.7
**O**	7.40	18.93	6.50	10.7	13.73	3.21	6.38	1.74	5.9	13.02	10.86	16.07	14.2	11.83	9.76	8.3
**Si**	0.50	1.62	1.24	4.19	1.30	1.14	1.39	0.00	0.35	5.19	2.75	2.19	2.8	0.25	0.74	1.76
**Cr**	0.10	0.29	0.57	0.77	0.56	0.25	0.27	0.00	0.11	0.17	0.5	0.32	0.45	0.06	0.08	0.85
**Fe**	0.54	39.16	2.18	1.86	1.56	0.67	0.60	0.35	0.35	0.75	1.48	0.83	1.3	0.18	0.43	2.07
**Ni**	6.06	21.37	79.18	60.54	69.87	77.05	78.89	3.75	7.55	20.44	65.04	21.34	36.08	3.04	5.96	69.86
**W**	77.81	2.77	2.98	13.4	2.59	11.61	2.51	81.79	69.51	44.16	17.06	49.74	27.07	78.76	75.4	9.47

The black residue consists of Ni-rich sphere droplets due to spattering (point 6), irregular Ni-rich particles (points 3, 4, 5, 7, 11, and 16), mono-crystalline WC particles (points 1, 8, 9, 14, and 15), and oxidized W–Ni–C particles (points 2, 10, 12, and 13). The existence of WC in the black residue is direct evidence of WC losses during the weld cladding process, resulting in lowered *f*_WC_ in the resultant overlay as compared to *f*_p_, as also observed by other studies of weld cladding of related overlays [23]. WC particles with no signs of melting and retaining their original shape show a very low concentration of Ni, indicating poor wettability of these particles with the molten droplet/pool. During deposition, some loose particles from the powder can fall and hit the weld pool. Depending on the impingement velocity of these particles, they might be unable to overcome the surface tension of the molten pool and therefore bounce off or sit on top of the molten pool surface and are further ejected by arc re-ignition [22]. It should be mentioned that all EDS measurements indicate high concentrations of oxygen, which is due to the oxidation of particles during the deposition process.

Guest [20, 22] illustrated the non-wetting behavior of WC by high-speed camera images during a standard GMAW process. They observed the non-wetting behavior of WC in all metal transfer modes and reported a minimum of 20% loss of WC because of this loss mechanism. In this work, the EDS results on the black residues indicate the existence of this loss mechanism in the CMT process as well. However, it should be pointed out that material transfer mode in CMT process is controlled by the mechanical detachment of droplets, which is fundamentally different from the standard GMAW process. The related behavior during CMT process will be investigated further in future studies using high-speed camera.

### Effect of processing parameters on the quality of Ni-WC MMC overlays

**Fig. 8:**
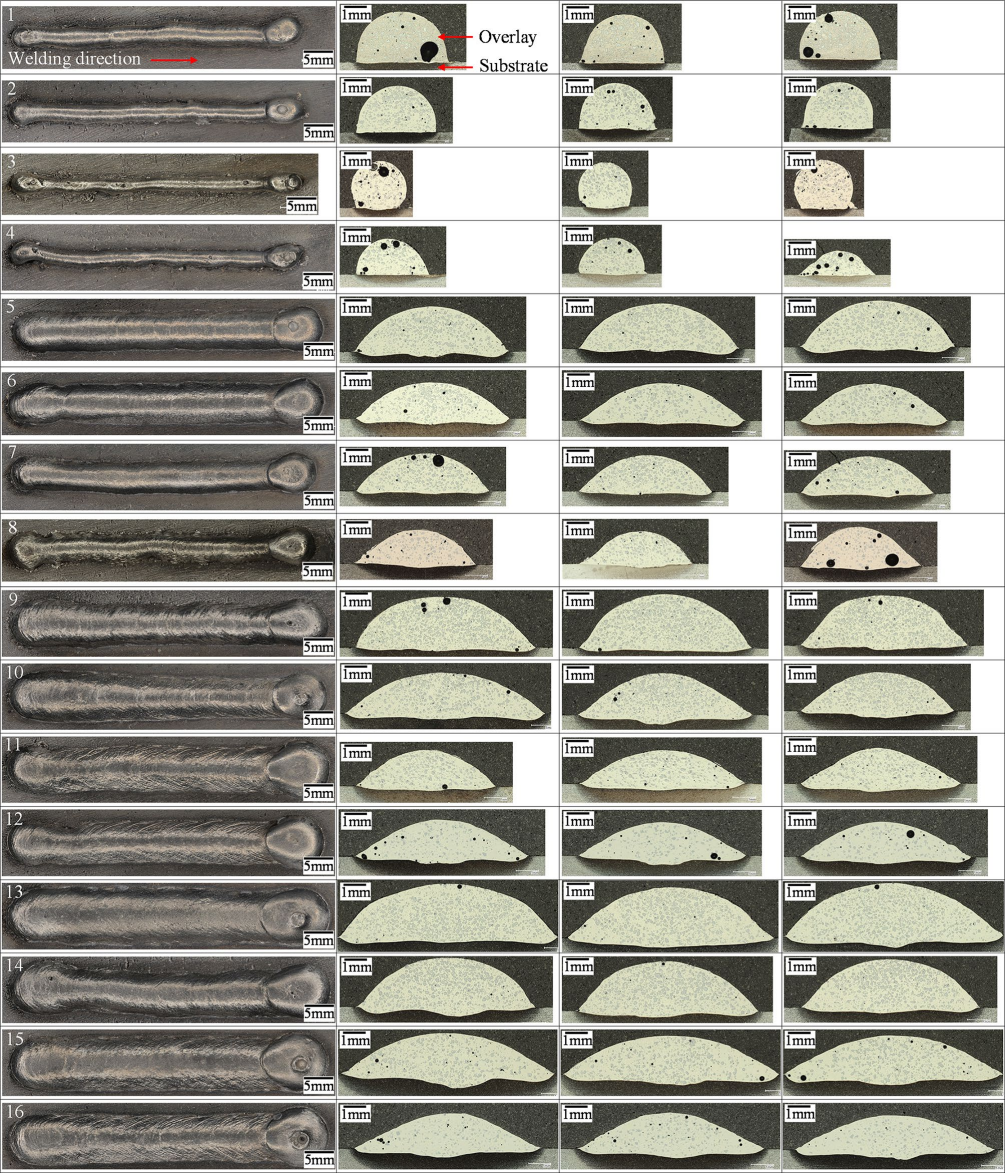
Top and cross-sectional views of the specimens

Figure 8 presents the top views and images of cross-sections of all single weld bead tracks. As can be seen, the bead profiles are continuous and uniform with low spatter. The cross-sections exhibit excellent metallurgical bonding to the substrates without crack, low porosity level, and relatively uniform distribution of the retained WC. These cross-sectional images provide the required information to determine *f*_WC_, *η*, and weld bead characteristics (*DL*, *P*, *H*, *W*, *W*/*H*, and *θ*), which are summarized in Table 9. The average porosity of all samples is 1.42%, while only four samples have the porosity greater than 2% (samples 1, 3, 4, and 8). The *HI* for these four samples is low (between 0.09 and 0.19 kJ/mm); sample 2 is also related to low *HI* (0.15 kJ/mm), but has low porosity level of 1%.

**Table 9:** Experimental results summarizing processing, carbide, and weld bead characteristics of all specimens

**ID**	***WFS*** **[m/min]**	***TS*** **[m/min]**	***SG*** **[%]**	***I***_**avg**_** [A]**	***V***_**avg**_** [V]**	***HI*** **[kJ/mm]**	***AIP*** **[kW]**	***f***_**WC**_ **[%]**	***η*** **[%]**	***DL*** **[%]**	***P*** **[mm]**	***DR*** **[kg/h]**	***W* [mm]**	***H* [mm]**	***W*/*H***	*θ* [°]
1	2.1	0.3	25% CO_2_	62.89	10.53	0.19	0.93	25.2	67.1	0.92	0.04	1.20	3.84	2.17	1.77	80
2	2.1	0.4	15% CO_2_	64.38	10.46	0.15	0.98	26.8	71.4	1.05	0.07	1.30	3.37	2.02	1.67	85
3	2.1	0.5	2% O_2_	64.37	8.97	0.10	0.85	20.7	55.5	1.65	0.09	1.28	1.92	2.06	0.93	122
4	2.1	0.6	5% O_2_	63.45	9.33	0.09	0.87	24.5	65.4	0.88	0.06	1.28	3.35	1.46	2.30	71
5	3.9	0.3	15% CO_2_	94.78	10.90	0.29	1.46	25.7	74.2	8.52	0.27	1.87	7.10	2.05	3.47	49
6	3.9	0.4	25% CO_2_	93.09	12.03	0.25	1.66	20.7	64.2	14.83	0.25	1.89	6.82	1.68	4.05	41
7	3.9	0.5	5% O_2_	91.24	9.82	0.16	1.36	23.0	65.2	6.77	0.15	2.01	6.00	1.67	3.60	50
8	3.9	0.6	2% O_2_	92.39	9.33	0.13	1.32	18.6	52.4	6.57	0.18	1.95	5.35	1.60	3.34	49
9	5.7	0.3	2% O_2_	127.78	9.46	0.35	1.75	27.5	78.0	6.93	0.19	2.40	7.70	2.28	3.37	49
10	5.7	0.4	5% O_2_	129.84	10.12	0.28	1.89	22.4	68.3	13.64	0.35	2.50	7.93	1.83	4.34	36
11	5.7	0.5	25% CO_2_	140.47	12.68	0.30	2.54	18.9	62.8	20.72	0.29	2.55	6.92	1.56	4.43	34
12	5.7	0.6	15% CO_2_	139.90	11.75	0.24	2.36	12.0	38.1	16.99	0.37	2.79	7.66	1.57	4.88	32
13	7.5	0.3	5% O_2_	166.34	10.30	0.47	2.34	26.3	77.4	10.08	0.42	3.03	9.86	2.40	4.11	43
14	7.5	0.4	2% O_2_	165.06	9.39	0.32	2.14	26.2	78.1	11.67	0.32	3.04	7.99	2.18	3.67	49
15	7.5	0.5	15% CO_2_	177.05	11.77	0.34	2.86	18.2	60.7	20.83	0.55	3.40	9.83	1.78	5.52	30
16	7.5	0.6	25% CO_2_	173.29	12.27	0.29	2.95	10.3	34.1	20.26	0.44	3.15	8.64	1.58	5.46	27

#### Effect of processing parameters on material transfers and weld bead characteristics

**Fig. 9:**
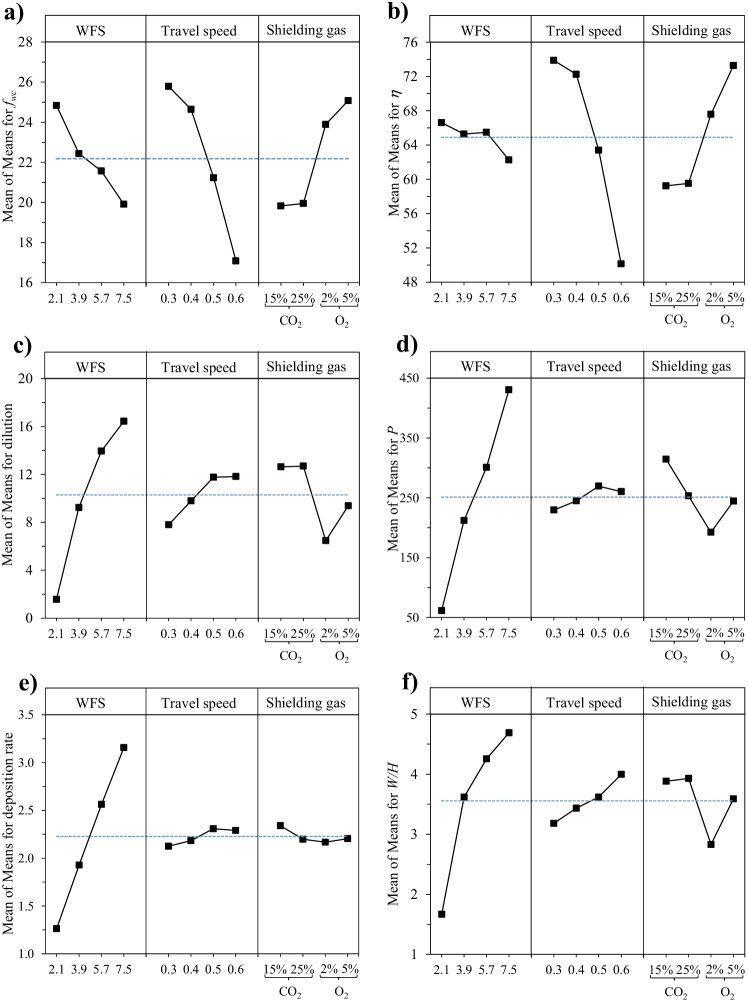
Main effect plots for **a** *f*_WC_; **b** *η*; **c** *DL*; **d** *P*; **e** *DR*; **f** *W/H* (with dash lines indicating means)

Figure 9a and b show the mean influence of processing parameters on *f*_WC_ and *η*, respectively. It can be seen that increasing the *WFS* and *TS* leads to a reduction of *f*_WC_ and *η*. Higher *WFS* involves higher current as indicated in Table 3, which increases *AIP* and thus arc energy, facilitating WC dissolution. The observation that increased *TS* leads to a reduction of *f*_WC_ and *η* is not in line with previous studies by other authors using standard GAMW processes, where higher *TS* was linked to lower *HI* and thus lower dissolution of WC [20, 46]; as discussed below, the effect of *TS* may be more complicated in the present study.

It should be noted that *η* is generally undervalued, depending on the level of WC dissolution. The concept for *f*_WC_ and *η* calculation is based on the assumption that all the retained WC in a weld bead track could be accounted for in the calculation. In reality, WC is inevitably partially dissolved. The image processing is arranged so that only retained WC and primary WC (formed during solidification) can be captured for *f*_WC_ and *η* evaluations. The capture of primary WC for *η* evaluations will compensate for the effect of dilution to a certain degree. However, all the secondary carbides are not accounted for by proper image processing, as they are mostly W-rich carbides containing Fe or Ni.

The level of CO_2_ in the SG mixture does not seem to have a significant effect on *f*_WC_ and *η*. However, *f*_WC_ and *η* are observed to increase with an increasing level of O_2_ in the *SG*. Improvements in *η* by O_2_ addition to the *SG* have previously been documented [22]. The addition of a controlled amount of oxidizing gas, such as CO_2_ or O_2_, to an Ar-rich *SG* mixture, can modify the thermophysical characteristics of plasma arc, improving welding arc stability, heat transfer, surface tension, and weldability [47–49]. Nevertheless, CO_2_ addition to the *SG* seems to be much less effective in achieving arc stabilization as compared to O_2_ addition for depositing the Ni-WC material system. Molten nickel resists oxidation, leading to fewer electron emissions and reducing stability at the cathodic locations. O_2_ addition in *SG* effectively promotes the oxidation of the nickel weld pool, improving arc stability during the droplet formation period [20]. Improved arc stability leads to increased *η* and, thus, increased *f*_WC_.

It can be observed in Fig. 9c–f that *WFS* is the main controlling factor for weld bead characteristics and *DR*. *WFS* determine *DR*, as it directly dictates the feeding rate of cored feed wire. Higher *WFS* also leads to higher arc energy, resulting in an increase in substrate melting (directly increasing *P*, *DL*, and *W*/*H*) and, thus, having a significant effect on weld bead characteristics. Higher *TS* also increases *DL*, *P*, and *W*/*H*, but its effect on *DR* is only marginal.

The effect of *TS* on *P* and *DL* can be understood by considering the combined effect of *HI* and arc impingement. According to Silva [50], *P* and *DL* are positively correlated with *HI*, but inversely correlated to impingement effect. At sufficient low *TS*, the impingement effect dominates, where the arc impinges on the weld pool rather than the substrate and the metal deposited acts as a barrier preventing direct arc impingement on the base metal; as such, lower *P* and *DL* are achieved at lower *TS* for the resultant overlay [30]. *P* and *DL* increase with increased *TS* until it reaches a critical point, after which the effect of *HI* dominates, and *P* and *DL* decrease with increased *TS*. As indicated in Fig. 9c and d, the critical *TS* could be around 0.5 m/min.

As shown in Fig. 9c and f, *DL* and *W*/*H* remain almost unchanged when using *SG* mixture with CO_2_ addition; however, O_2_ addition in the SG results in relatively lower *DL* and *W*/*H*. This is due to the change of thermophysical characteristics of the plasma arc, resulting in reduced surface tension of the weld pool, enhancing the wettability of the weld pool and heat transfer, leading to shallower *P* (i.e., lower *DL*), and altering the weld bead geometry [47–49]. SG compositions do not show any significant effect on the *DR*, as seen in Fig. 9e.

**Fig. 10:**
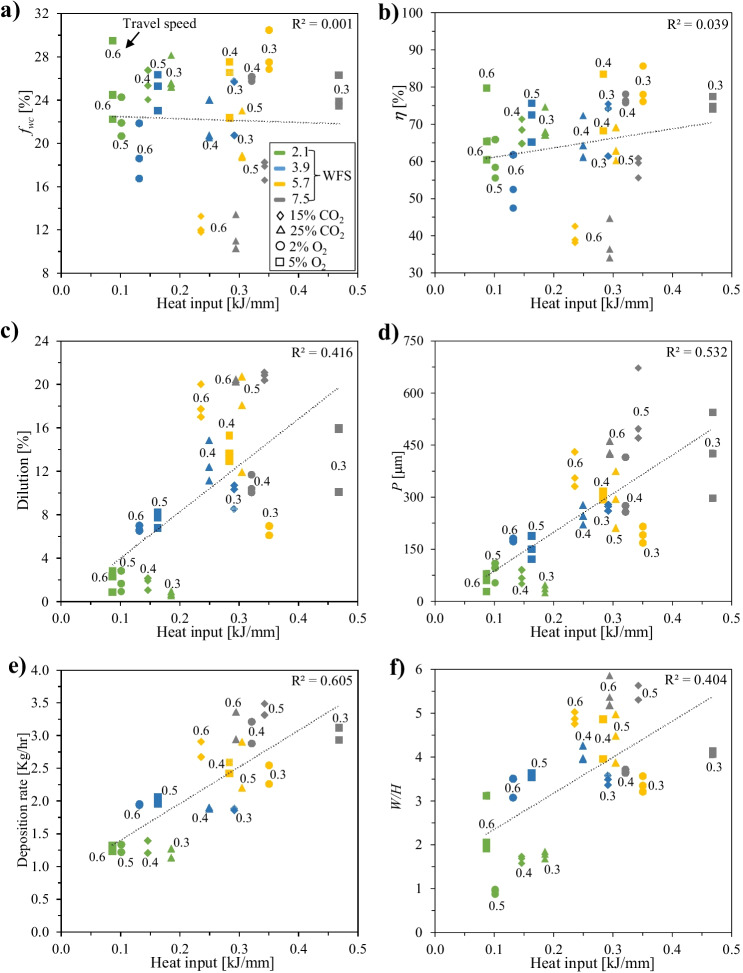
Effect of *HI* on quality criteria: **a** *f*_WC_; **b ***η*; **c** *DL*; **d** *P*; **e** *DR*; **f** *W*/*H*

**Fig. 11:**
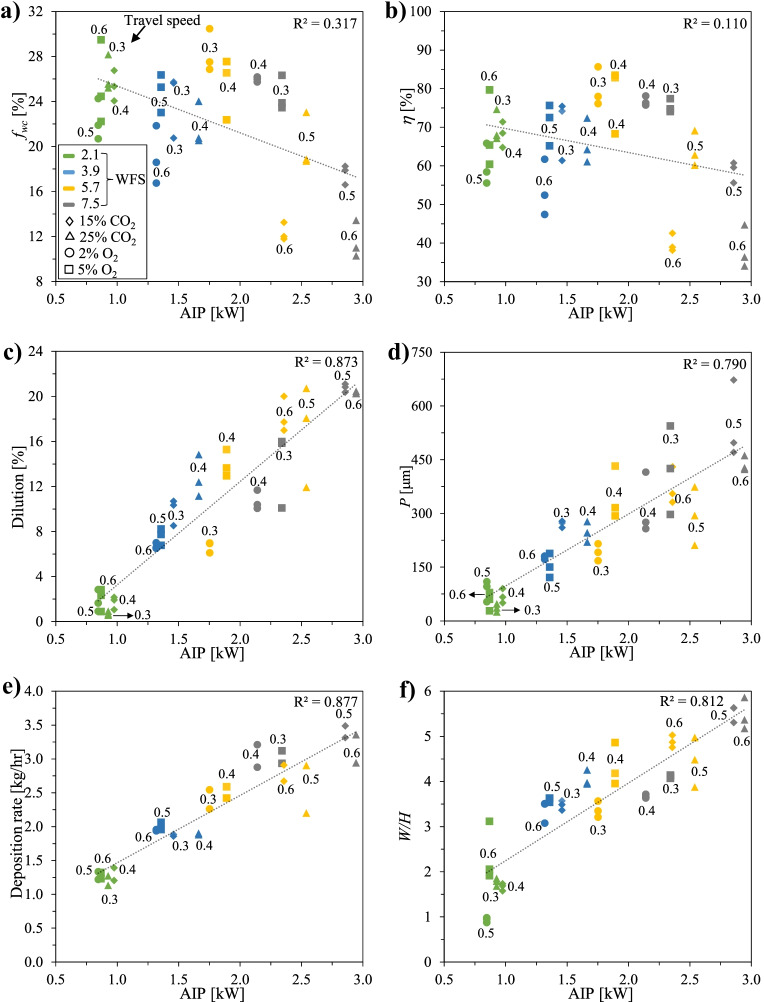
Effect of *AIP* on quality criteria: **a** *f*_WC_; **b ***η*; **c** *DL*; **d** *P*; **e** *DR*; **f** *W*/*H*

Figures 10 and 11 illustrate the effect of *HI* and *AIP* on *f*_WC_, *η*, *DR*, and weld bead characteristics, respectively. The data in these graphs are fitted by a linear function; *R*^2^ (shown in each plot) is a measure of overall closeness of the data to the fitted regression line; the larger the *R*^2^ value (between 0 and 1), the better the linear regression fitting. No meaningful correlation can be identified between *f*_WC_/*η* and *HI*/*AIP*, which is in contrast to the observation for standard GMAW processes where *f*_WC_ is negatively correlated with *HI* [11, 51]. It seems that the most important factor for determining *f*_WC_ or *η* in the current study is the *TS*, with *WFS* being the second most important factor, as indicated in Fig. 9. Although *HI* or *AIP* varies with changing *WFS* for a CMT synergic mode, their effects on *f*_WC_ or *η* may not be fully interconnected, as other factors (such as *TS*) also play a role here. It is interesting to notice that high level of *f*_WC_ or *η* can be accomplished even at high *HI*, where high *DR* and good weld bead appearance are also achieved. Vespa et al. [18] indicated a similar trend using controlled short-circuit GMAW process, which is comparable to the CMT process but with less complex waveform control.

As shown in Figs. 10c–f and 11c–f, *DR*, *DL*, *P*, and *W*/*H* exhibit, in general, a positive correlation with *HI* or *AIP*, respectively. This correlation seems to be more pronounced for *AIP* as compared to *HI*, which was observed by other researchers as well [30, 52]. Moreover, the results also support the assumption that *AIP* can be a better governing factor to control weld bead characteristics and *DR* than *HI*, as suggested by other authors [19, 52].

**Fig. 12:**
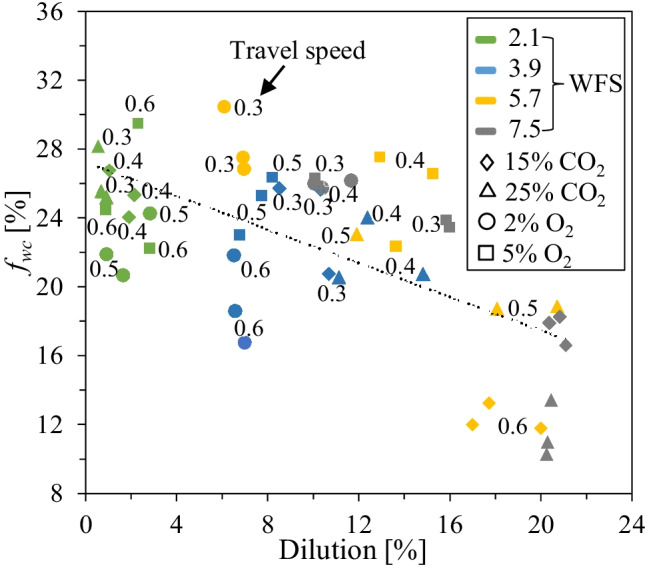
Effect of *DL* on *f*_WC_

As indicated in Fig. 12, a generally negative correlation can be identified between *DL* and *f*_WC_. The results suggest that *f*_WC_ of above 20% are achieved at *DL* below 5%. However, in cases of higher *DL* (> 15%) related to high *WFS* (5.7–7.5 m/min) and high *TS* (0.6 m/min), the amount of *f*_WC_ is drastically reduced. The same behavior was reported by Günther and Bergmann [46], who showed that high *DL* is directly related to the higher concentration of Fe migrated into the overlay from the substrate, leading to the excessive dissolution of WC.

#### Effect of processing parameters on microstructures

**Fig. 13:**
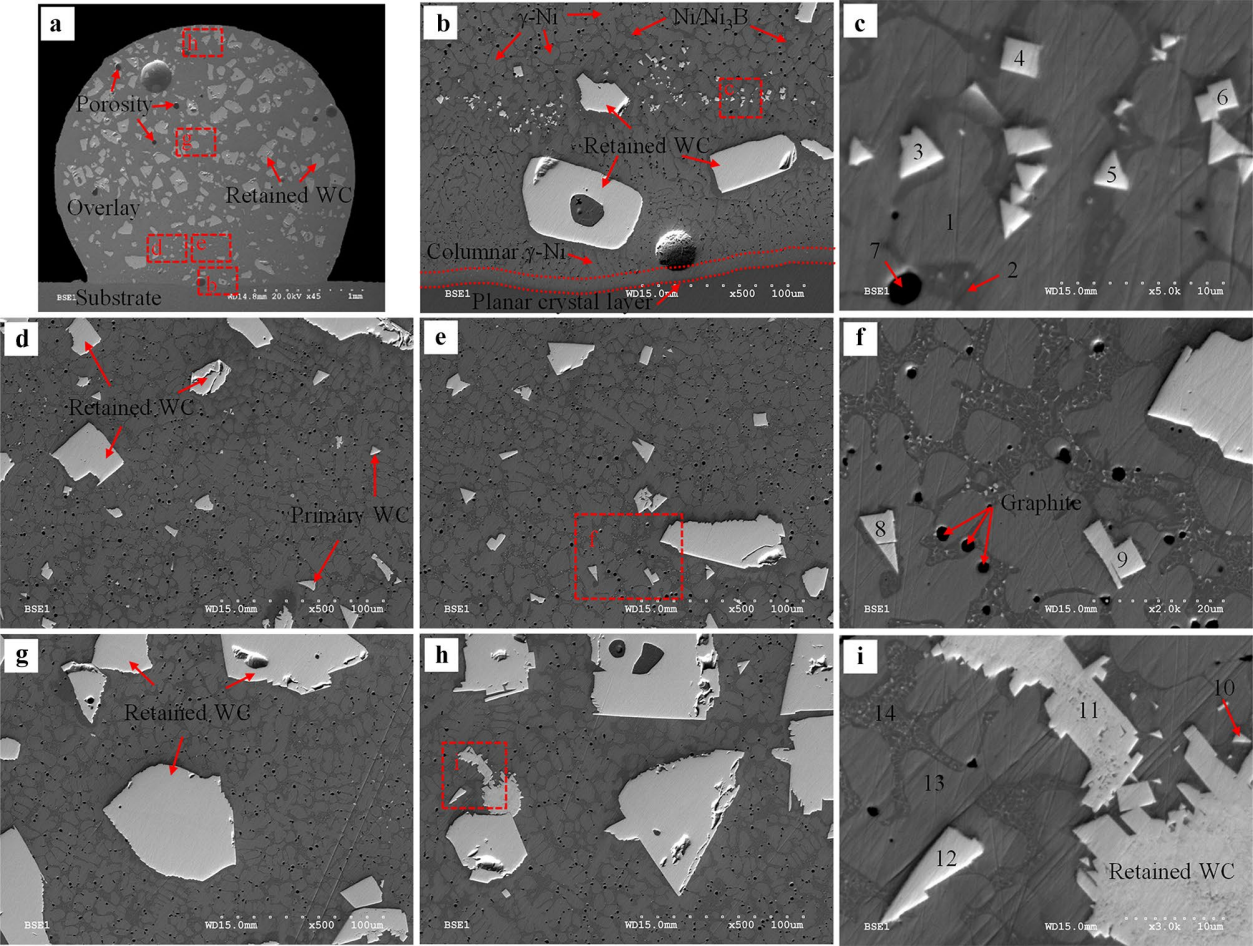
BSE images of sample 3 (*AIP*: 0.85 kW): **a** overlay cross-section; **b** interface of the substrate/overlay; **c** higher magnification of the selected area in **b**; **d** and **e** lower region; **f** higher magnification of the selected area in **e**; **g** middle region; **h** upper region; **i** higher magnification of the selected area in **h**

**Fig. 14:**
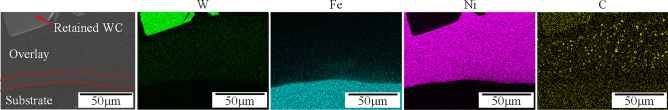
EDS map of the interface of substrate/overlay of sample 3 (*AIP*: 0.85 kW)

**Fig. 15:**
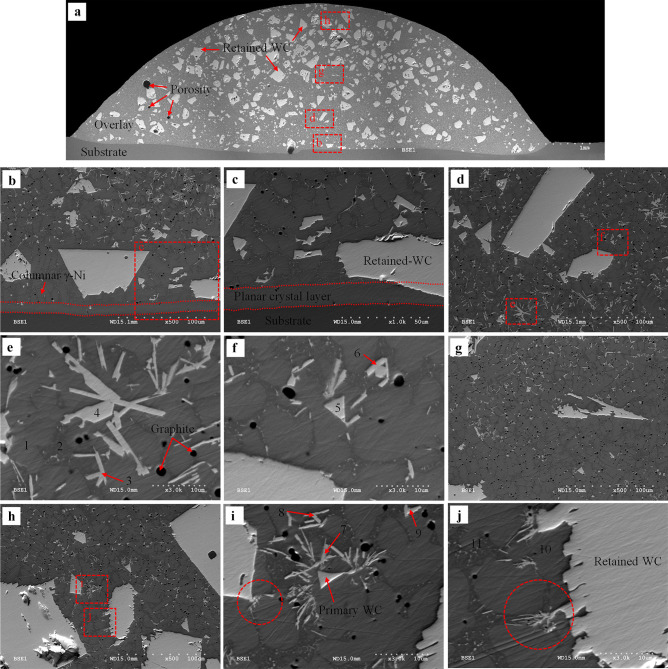
BSE images of sample 7 (*AIP*: 1.36 kW): **a** whole overlay cross-section; **b** interface of the substrate/overlay; **c** higher magnification of the selected area in **b**; **d** lower region; **e** and **f** higher magnification of the selected area in **d**; **g** middle region; **h** upper region; **i** and **j** higher magnification of the selected area in **h**

**Fig. 16:**
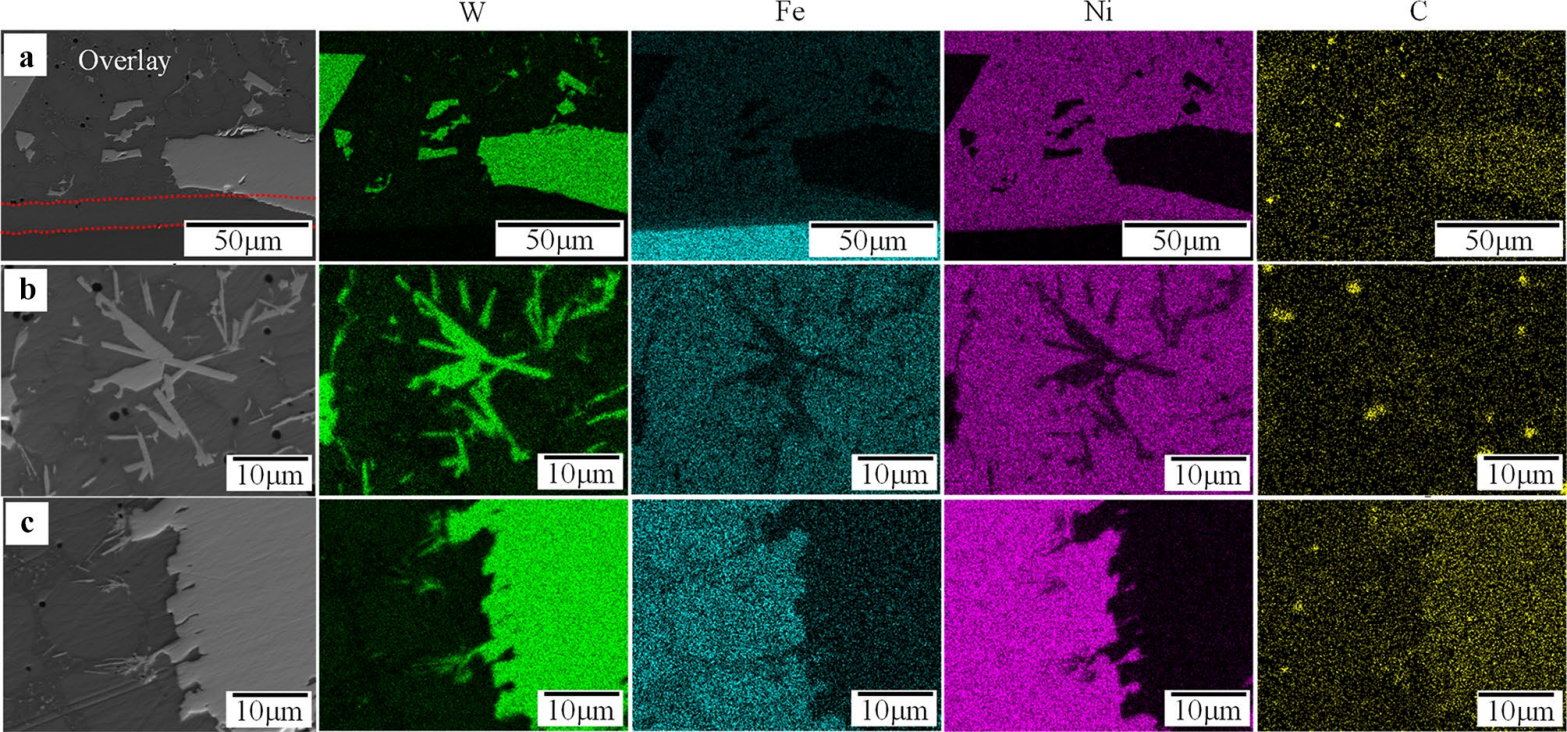
EDS map of the cross-section of sample 7 (*AIP*: 1.36 kW): **a** interface of substrate/overlay; **b** secondary carbide at lower region; **c** selected area at upper region

**Fig. 17:**
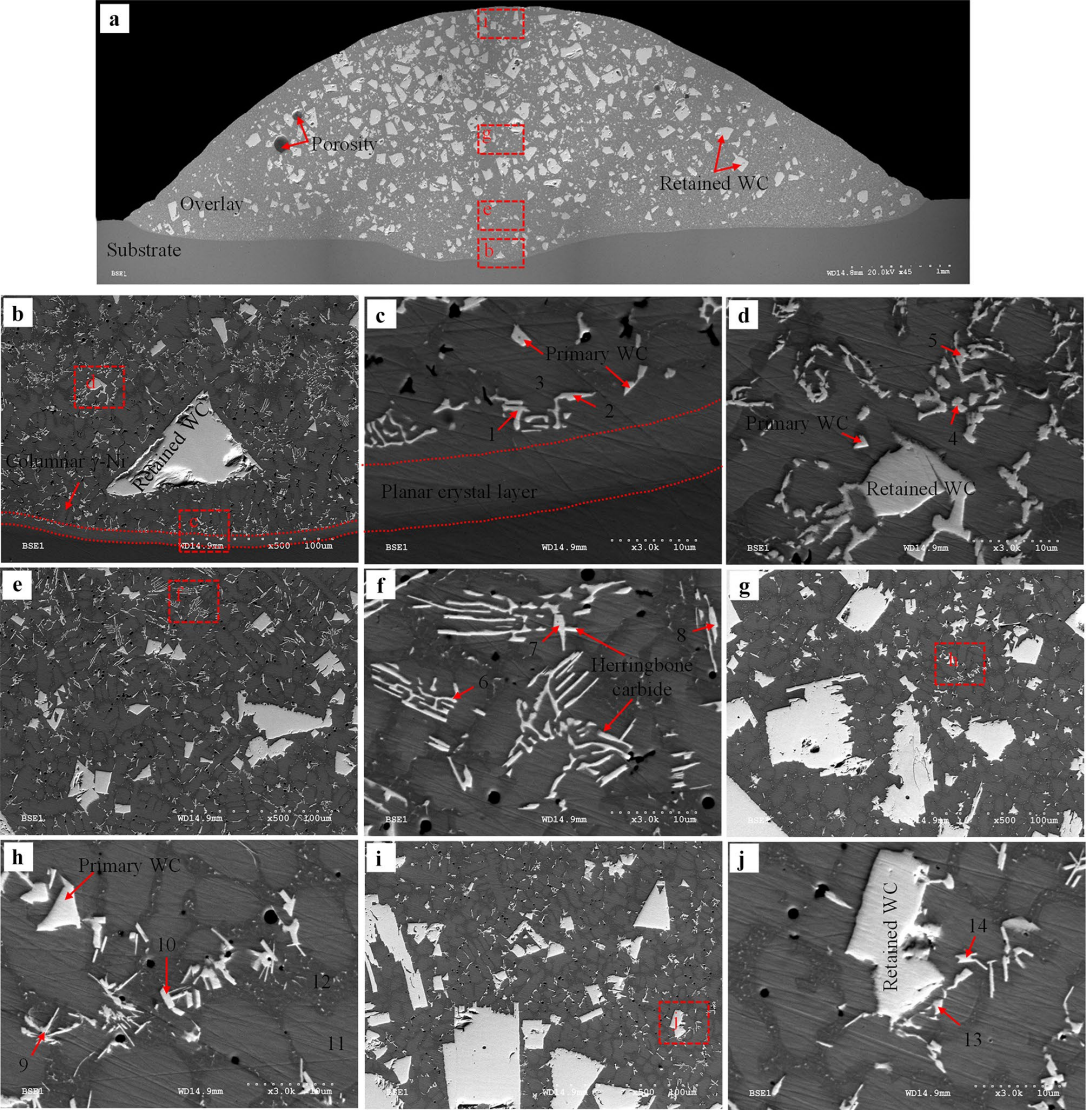
BSE images of sample 10 (*AIP*: 1.89 kW): **a** overlay cross-section; **b** interface of the substrate/overlay; **c** and **d** higher magnification of the selected area in **b**; **e** lower region; **f** higher magnification of the selected area in **e**; **g** middle region; **h** higher magnification of the selected area in **g**; **i** upper region; **j** higher magnification of the selected area in **i**

**Fig. 18:**
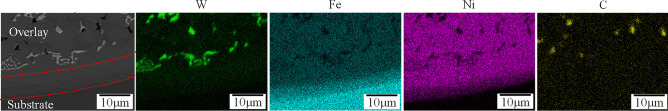
EDS map of the interface of substrate/overlay of sample 10 (*AIP*: 1.89 kW)

**Fig. 19:**
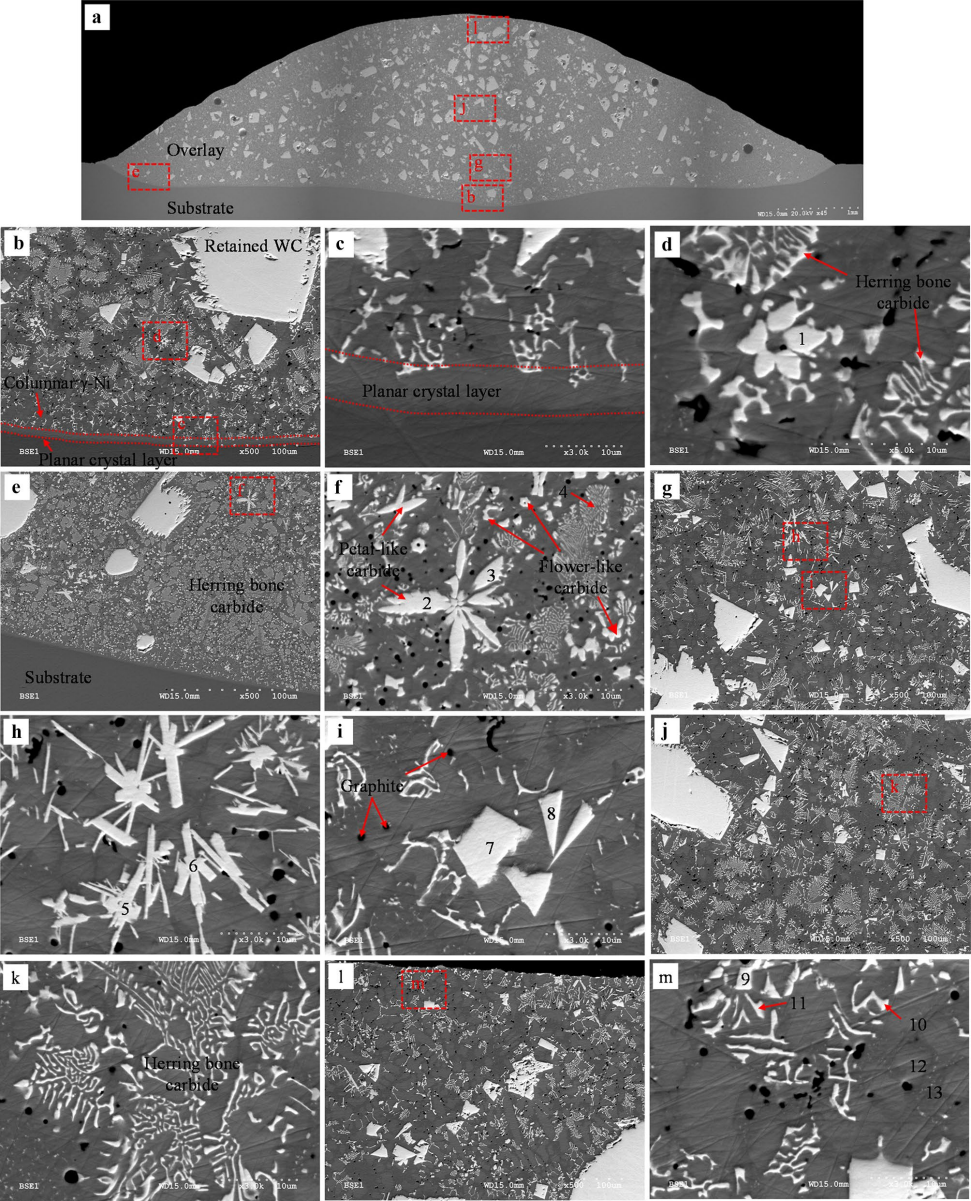
BSE images of sample 16 (*AIP*: 2.95 kW): **a** overlay cross-section; **b** interface of the substrate/overlay; **c** and **d** higher magnification of the selected area in **b**; **e** left toe interface; **f** higher magnification of the selected area in **e**; **g** lower region; **h** and **i** higher magnification of the selected area in **g**; **j** middle region; **k** higher magnification of the selected area in **j**; **l** upper region; **m** higher magnification of the selected area in **l**

Samples 3, 7, 10, and 16 were analyzed to evaluate the effect of processing parameters on the microstructure; the related *AIP* for deposition of these specimens is 0.85, 1.36, 1.89, and 2.95 kW, respectively. Figures 13, 14, 15, 16, 17, 18, and 19 show the micrographs of these samples taken from the whole overlay area. EDS spot measurement results of the selected areas are presented in Tables 10, 11, 12, and 13.

**Table 10:** EDS spot results of marked locations in Fig. 13

Elements (wt.%)	Point													
	**1**	**2**	**3**	**4**	**5**	**6**	**7**	**8**	**9**	**10**	**11**	**12**	**13**	**14**
**C**	7.48	6.10	19.84	20.48	18.67	20.79	51.15	14.71	19.47	20.62	19.78	19.31	4.44	4.58
**O**	0.00	0.00	0.11	0.23	0.11	0.00	0.00	0.37	0.00	0.00	0.01	0.13	0.00	0.00
**Si**	0.00	0.00	0.00	0.00	0.00	0.00	0.02	0.00	0.00	0.00	0.00	0.00	0.00	0.59
**Cr**	0.99	0.56	1.49	1.63	1.48	0.99	0.56	1.33	1.75	3.66	0.09	1.27	0.98	2.23
**Fe**	4.92	5.31	0.28	0.37	0.54	0.35	3.57	0.28	0.41	0.3	0.17	0.19	1.39	1.30
**Ni**	73.94	73.02	6.40	7.75	13.5	8.73	42.00	4.3	9.54	15.4	3.2	5.67	75.53	77.49
**W**	12.67	15.01	71.88	69.54	65.7	69.14	2.71	79.00	68.83	60.03	76.75	73.42	17.66	13.81

**Table 11:** EDS point results of marked locations in Fig. 15

Elements (wt.%)	Point										
	**1**	**2**	**3**	**4**	**5**	**6**	**7**	**8**	**9**	**10**	**11**
**C**	4.31	5.87	15.68	17.00	13.65	11.75	16.45	16.41	15.91	3.57	3.99
**O**	0.18	0.00	0.00	0.00	0.36	0.02	0.00	0.00	0.00	0.00	0.00
**Si**	0.01	0.30	0.00	0.00	0.00	0.00	0.00	0.00	0.00	0.16	0.36
**Cr**	0.83	1.93	1.99	0.93	2.05	3.83	3.14	4.00	3.52	0.98	1.48
**Fe**	13.60	11.57	3.80	2.52	2.67	3.81	2.70	2.18	2.22	9.00	7.92
**Ni**	65.98	61.29	15.88	11.58	7.58	12.19	16.69	13.56	14.96	72.25	74.19
**W**	15.09	19.03	62.66	67.98	73.68	68.4	61.02	63.85	63.39	14.05	12.06

**Table 12:** EDS point results of the marked locations in Fig. 17

Elements (wt.%)	Point													
	**1**	**2**	**3**	**4**	**5**	**6**	**7**	**8**	**9**	**10**	**11**	**12**	**13**	**14**
**C**	10.17	6.35	3.78	15.12	16.16	16.74	17.71	15.89	18.17	19.21	2.9	3.11	16.5	16.29
**O**	0.17	0.03	0.44	0.00	0.00	0.00	0.00	0.00	0.00	0.00	0.18	0.00	0.20	0.45
**Si**	0.00	0.17	0.15	0.00	0.00	0.00	0.00	0.25	0.00	0.00	0.31	0.45	0.00	0.00
**Cr**	0.83	1.02	0.66	1.06	0.90	1.23	1.39	1.41	3.46	3.92	0.87	1.19	3.32	3.24
**Fe**	21.46	23.3	24.60	7.78	8.07	8.87	7.98	9.56	3.67	2.78	16.16	13.55	2.96	2.80
**Ni**	49.88	62.45	61.50	47.3	47.59	36.14	29.56	45.74	13.34	7.01	67.34	73.51	10.55	7.69
**W**	17.48	6.67	8.87	28.74	27.27	37.02	43.36	27.15	61.36	67.08	12.24	8.19	66.47	69.52

**Table 13:** EDS point results of marked locations in Fig. 19

Elements (wt.%)	Point												
	**1**	**2**	**3**	**4**	**5**	**6**	**7**	**8**	**9**	**10**	**11**	**12**	**13**
**C**	16.61	13.92	13.82	7.63	15.73	15.32	19.88	18.41	15.35	16.46	15.86	5.58	6.68
**O**	0.00	0.41	0.27	0.15	0	0.02	0.37	0.29	0.74	0.17	0.07	0.26	0.04
**Si**	0.00	0.00	0.00	0.00	0.00	0.00	0.00	0.00	0.00	0.00	0.00	0.03	0.53
**Cr**	1.10	1.38	1.45	0.95	1.11	0.87	0.85	1.22	1.26	1.39	1.13	0.68	1.14
**Fe**	5.67	4.51	4.19	17.86	6.57	4.87	1.55	1.60	1.79	8.16	11.06	22.54	22.31
**Ni**	12.49	7.88	7.08	41.35	15.36	11.88	2.79	2.58	2.94	17.44	25.39	58.53	58.86
**W**	64.13	71.91	73.19	32.07	61.22	67.04	74.56	75.89	77.92	56.38	46.49	12.38	10.44

##### Microstructure characterization of sample 3 (AIP: 0.85 kW)

The matrix is composed of a light gray phase (e.g., points 1 and 13 in Fig. 13) and a dark gray phase (e.g., points 2 and 14). EDS results show a high concentration of Ni, varying from 73.94 to 77.49 wt.%, see Table 10. The matrix microstructure is similar to a conventional Ni-based matrix microstructure, as also indicated by Liyanage et al. [9]; hence, the light and dark gray phases are characterized as γ-Ni dendrites and γ-Ni + Ni_3_B interdendritic eutectic region, respectively. Because of the higher temperature gradient near the interface between the substrate and overlay [53, 54], the planar crystal layer is formed first near the interface, followed by columnar γ-Ni dendrites along the solidification path, as illustrated in Fig. 13a and b. The EDS maps of the substrate/overlay interface show a visible diffusion zone of migrated Fe from the substrate into the overlay, indicating a good metallurgical bond (see Fig. 14). By moving towards the top of the overlay, the concentration of Fe gradually decreases, as confirmed by EDS results (see Table 10) with Fe concentration of 4.92–5.31 wt.% at the interface (points 1 and 2) and 1.3–1.39 wt.% (points 13 and 14) at the top of the overlay.

A degradation seam is not observed around the retained WC particles. They show an excellent metallurgical bond to the adjacent matrix, in contrast to the cases with retained WC/W_2_C eutectic particles around which a degradation seam usually forms due to preferential dissolution of W_2_C (as W_2_C is less stable than WC) [19, 55]. Nevertheless, the mono-crystalline WC particles can inevitably partially dissolve in the molten pool during deposition, increasing the concentration of W in the Ni-based matrix (see Table 10, points 1, 2, 13, and 14).

Small precipitates are found within the γ-Ni dendrites (see Fig. 13b and c). These homogeneously formed precipitates (< 15 μm) have triangle and rectangle morphologies presence throughout the whole thickness of the overlay, as also observed by Zhao et al. [55]. EDS analysis results on these precipitates (see Table 10, points 3–6) indicate high W and C concentrations ranging from 69 to 72 wt.% and 18 to 21 wt.%, respectively; therefore, these tiny particles can be identified as primary WC precipitates formed from free W and C (due to dissolution of WC particles) in the molten pool during the solidification process, as also reported in previous literature [16, 18, 42, 55]. The formation of primary WC can be realized by considering the C-rich side of the Ni–W–C ternary phase diagram [56], where WC grains develop into a three-dimensional triangular prism via a layer-by-layer growth mechanism. Random cross-sectioning of primary WC triangular prism [57] is responsible for the different morphology observed, varying from equilateral triangles, isosceles triangles, and irregular triangles to quadrilaterals [42].

Black semi-spherical precipitates are observed in Fig. 13c and f. EDS result at point 7 reveals that this precipitate is rich in carbon. The same black semi-spherical precipitates were reported in laser cladding deposition of Ni-WC MMCs on carbon steel [16] or cast iron substrate [58]. Using bright field transmission electron microscopy, Zhao et al. [16] characterized these precipitates as incompletely graphitized residual carbon formed due to the rapid solidification process. According to the Ni–C binary phase diagram [59], there is only a limited solubility of carbon into nickel, and γ-Ni + graphite + WC three-phase can be thermodynamically stable at certain elevated temperatures [18, 56]. Therefore, under high cooling rates, graphite precipitates could be retained in the overlay at room temperature [16, 18, 58].

As shown in Fig. 13d–i, moving from the lower to the upper region of the overlay, the microstructure remains relatively unchanged; this may be due to lower *HI* (resulting in higher thermal gradient and faster solidification rate), which suppresses overall phase transformation at low temperatures, enabling metastable phases retained at the room temperature. EDS results indicate that points 8–12 are WC. The microstructure consists of the retained WC, γ-Ni dendrites, γ-Ni + Ni_3_B lamellar eutectic in the interdendritic region, primary WC, and graphite precipitates.

##### Microstructure characterization of sample 7 (AIP: 1.36 kW)

For sample 7, the matrix is also composed of γ-Ni dendrites (points 1 and 10 in Fig. 15) and γ-Ni + Ni_3_B lamellar eutectic in the interdendritic region (points 2 and 11). Similar to sample 3, the substrate/overlay interface is identified by the planar crystal layer followed by columnar γ-Ni dendrites formed along the solidification path (see Fig. 15b and c). The EDS maps of the substrate/overlay interface show a visible diffusion zone of migrated Fe from the substrate into the overlay, indicating a good metallurgical bond (see Fig. 16a). According to Table 11 (EDS point results of marked locations in Fig. 15), the amount of dissolved Fe is 11.57–13.6 wt.% at the interface (points 1 and 2) and 7.92–9 wt.% (points 11 and 12) at the top of the overlay. Because of the increased *DL*, there is more dissolved Fe in sample 7 than in the matching regions of sample 3. The black precipitates are graphite uniformly distributed throughout the overlay.

Homogeneously formed precipitates of primary WC (i.e., point 5 in Fig. 15f) are detected. Moving from the lower to the upper region of the overlay, the formation of secondary W-rich carbides with the rod/grass-like (points 3, 6, and 7–9) and blocky (point 4) shapes are also detected, as shown in Fig. 15d–j. In reference to Zhao et al. [16], considering EDS analyses and the morphology of these carbides, the rod/grass-like ones may be identified as WC while the blocky ones as W_2_C. The retained WC can provide heterogeneous nucleation sites for the secondary carbides, which may be promoted by the locally excessive W and C owing to partial WC dissolution; the secondary carbides can preferentially grow along the edges of the retained WC particles (see the marked area in Figs. 15i and j and 16c). Formation of these new phases and morphologies can also be realized due to higher *HI* and lower cooling rate [54].

##### Microstructure characterization of sample 10 (AIP: 1.89 kW)

Sample 10 has a different microstructure than samples 3 and 7 due to higher *HI*, *AIP*, and *DL*, leading to a lower cooling rate which facilitates the formation of secondary W-rich carbides, as shown in Fig. 17. The main difference may be the formation of herringbone M_6_C carbide at the interface, lower and middle region of the overlay. According to the EDS results (Fig. 18 and Table 12) and studies by other researchers [16, 42], points 1 and 2 can be characterized as Fe_3_W_3_C and points 6–8 as Ni_2_W_4_C. Furthermore, the diffusion zone at the substrate/overlay interface is wider than samples 3 and 7. EDS result of point 3 shows a higher concentration of dissolved Fe at a level of 24.6 wt.% (see Table 12), consistent with its higher *DL*. The microstructure at the middle to the upper region of the overlay matrix mainly consists of γ-Ni dendrites, γ-Ni + Ni_3_B lamellar eutectic, newly formed WC with triangles/quadrilaterals or rod/grass-like morphology (points 9, 10, 13, and 14).

##### Microstructure characterization of sample 16 (AIP: 2.95 kW)

**Fig. 20:**
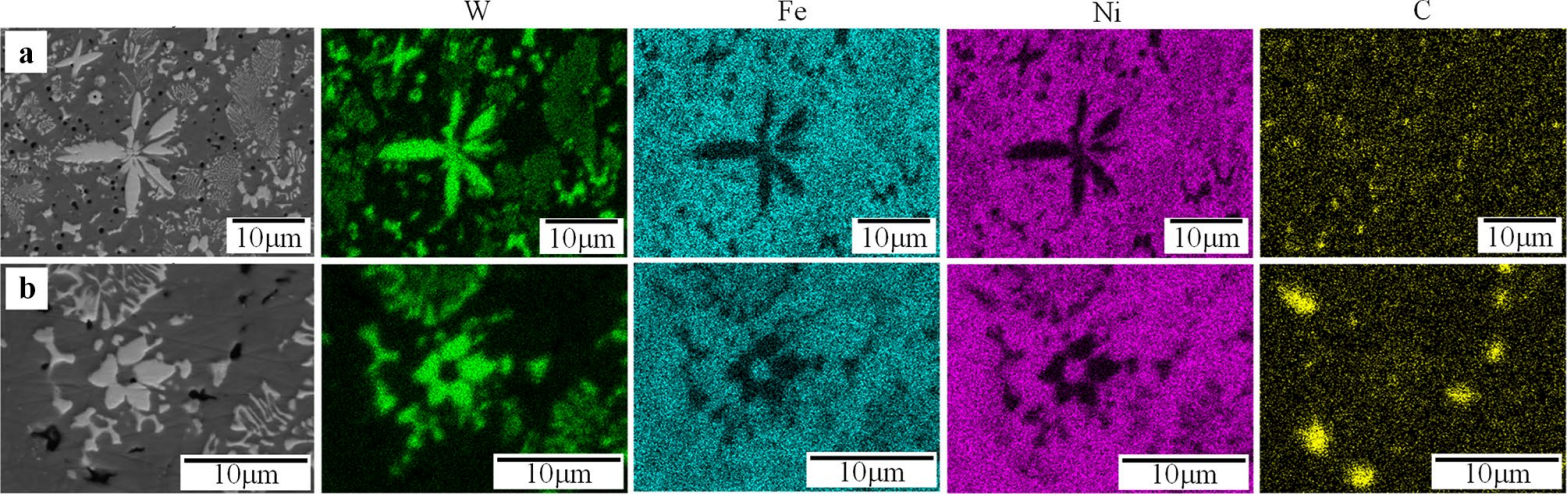
EDS map of secondary carbide of sample 16 (*AIP*: 2.95 kW) from selected area

Sample 16 shows a notable microstructural difference from other characterized samples due to its highest *DL* and *AIP* (resulting in the lowest thermal gradient and cooling rate), which facilitates the dissolution of WC in the molten pool and solid phase transformation during the cooling process. Apart from the detected microstructure discussed in previous samples, secondary carbides are observed with various additional morphologies, including herringbone, flower-like, and petal-like ones, as shown in Fig. 19. Herringbone precipitates formed throughout the whole overlay, which is attributed to the higher dwell time of the molten pool. According to the EDS results (Fig. 20 and Table 13), point 4 may be identified as Fe_3_W_3_C and points 9–11 likely as Ni_2_W_4_C. Furthermore, flower and petal-like carbides (points 1–3) are rich in W and are likely W_2_C [60]. Primary WC is observed as rod-like precipitates (points 5 and 6) or triangles/quadrilaterals (points 7 and 8). The formation of Fe_3_W_3_C is plausible even in the upper region of the overlay due to available Fe at a level of 22.31 to 22.51 wt.% in the matrix (see points 12 and 13 in Table 13).

### Recommended processing parameters for overlay quality improvement

**Fig. 21:**
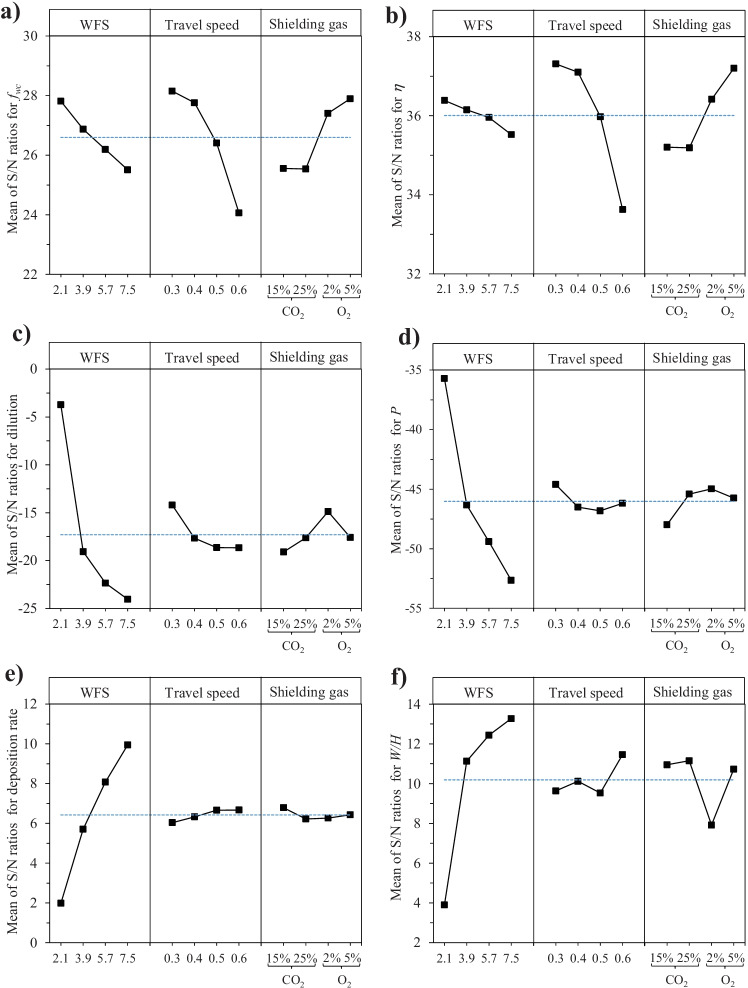
Main effects plots of S/N ratio for **a** *f*_WC_; **b** *η*; **c** *DL*; **d** *P*; **e** *DR*; **f** *W*/*H* (with dash lines indicating means of the S/N ratios)

Figure 21 and Table 14 show the main effect plots of the S/N ratio for *f*_WC_, *η*, *DL*, *P*, *DR*, and *W*/*H*, in accordance with the respective goal of the experiments (see Table 6). In general, higher S/N ratio values suggest processing parameters that reduce the impact of noise components and variability and improve performance [61].

**Table 14:** S/N ratio results of the experiments

ID	*WFS* [m/min]	*TS* [m/min]	*SG* [%]	S/N ratio [dBi]					
				*f*_WC_	*η*	*DL*	*P*	*DR*	*W*/*H*
1	2.1	0.3	25% CO_2_	28.37	36.86	2.63	− 31.39	1.55	4.94
2	2.1	0.4	15% CO_2_	28.06	36.65	− 4.95	− 36.98	2.19	4.40
3	2.1	0.5	2% O_2_	26.90	35.49	− 5.87	− 39.01	2.09	− 0.63
4	2.1	0.6	5% O_2_	27.92	36.54	− 6.74	− 35.49	2.10	6.89
5	3.9	0.3	15% CO_2_	27.48	36.83	− 19.91	− 48.64	5.44	10.81
6	3.9	0.4	25% CO_2_	26.69	36.31	− 22.20	− 47.92	5.53	12.15
7	3.9	0.5	5% O_2_	27.87	36.98	− 17.62	− 43.83	6.05	11.12
8	3.9	0.6	2% O_2_	25.45	34.47	− 16.52	− 44.98	5.78	10.48
9	5.7	0.3	2% O_2_	28.99	38.02	− 16.49	− 45.68	7.56	10.54
10	5.7	0.4	5% O_2_	28.01	37.75	− 22.91	− 50.94	7.96	12.63
11	5.7	0.5	25% CO_2_	25.99	36.08	− 24.76	− 49.56	7.89	12.81
12	5.7	0.6	15% CO_2_	21.79	31.98	− 25.24	− 51.47	8.88	13.77
13	7.5	0.3	5% O_2_	27.77	37.54	− 23.07	− 52.75	9.61	12.27
14	7.5	0.4	2% O_2_	28.29	37.69	− 20.61	− 50.20	9.63	11.29
15	7.5	0.5	15% CO_2_	24.87	35.34	− 26.35	− 54.86	10.62	14.83
16	7.5	0.6	25% CO_2_	21.10	31.51	− 26.16	− 52.82	9.91	14.72

Table 15 lists the “best” parameters, independently selected based on S/N ratio and experiment goal for each quality criterion without considering interaction with other criteria. It can be seen that the recommended processing parameters to improve *f*_WC_, *η*, *DL*, and *P* are opposite to what is required for improving *DR* and *W*/*H*. Therefore, it is difficult to find optimum processing parameters to meet all quality requirements; trade-offs are needed in engineering practices based on specific needs.

**Table 15:** Taguchi's recommended processing parameters for overlay quality improvement

Quality criterion	Recommended process control parameter				Remark
	*WFS* [m/min]	*TS* [m/min]	*SG* [%]	Predicted S/N [dBi]	
*f*_WC_	2.1	0.3	5% O_2_	30.66	The recommended parameters are disadvantageous for deposition rate and *W*/*H*
*η*	2.1	0.3	5% O_2_	38.89	
*DL*	2.1	0.3	2% O_2_	1.78	
*P*	2.1	0.3	2% O_2_	− 33.24	
*DR*	7.5	0.6	15% CO_2_	10.24	Disadvantageous for *f*_WC_, *η*, *DL*, and *P*
*W***/***H*	7.5	0.6	25% CO_2_	15.58	

In weld cladding of Ni-WC MMC to steel substrates, keeping the *DL* below 10% is recommended to ensure a high-quality overlay [62]. Abioye et al. [63] proposed *DL* between 5 and 13% with *θ* less than 80° for Ni-WC MMC. As indicated by Choi et al. [4], *DL* below 1% is inadequate for oil-sand applications, particularly those involving mechanical impact loading. Moreover, Zhao et al. [44] suggested a *DL* of at least 1% for a satisfactory metallurgical bond between the substrate and the overlay. Hence, considering our targeted applications, it is rational to aim the *DL* of 1–10%. At this *DL* range (1–10%), the average *f*_WC_ in our experiments varied between 19 and 28%, as shown in Fig. 12. Furthermore, *W/H* greater than two is recommended for weld cladding to avoid a lack of fusion in subsequent passes [50, 64, 65].

It is known that *f*_WC_ and *η* are the most significant quality factors for Ni-WC MMC overlays compared to other criteria. On that basis, it seems that *WFS* at 2.1 m/min should be the best choice; however, weld cladding with *WFS* at 2.1 m/min could result in *W*/*H* smaller than two, as is the case for experiment runs 1–3 (see Fig. 9 and Table 9). The next in line is *WFS* of 3.9 m/min, which will keep the *DL* under 10% and can significantly improve the *DR* and *W*/*H*, but does not cause a drastic reduction in *f*_WC_ and *η* (see Fig. 9). As to the *SG* mixture, the one with 5% O_2_ addition seems to be the best choice to improve *f*_WC_ and *η*.

### Confirmation tests

**Fig. 22:**
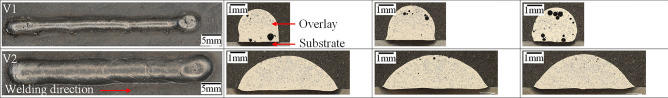
Top and cross-sectional views of confirmation test samples

Based on the deliberations in Section 3.4 and in reference to Table 15, *TS* of 0.3 m/min, *SG* with 5% O_2_ addition, and *WFS* of 2.1 and 3.9 m/min are selected for the two intended confirmation tests (as listed in Table 16). Figure 22 illustrates the top view and transverse cross-sectional images of the resultant weld bead tracks, showing excellent metallurgical bonding to the substrate without crack. The values of *f*_WC_, *η*, *DL*, and weld bead geometry (*P*, *W*/*H*, and *θ*) are summarized in Table 17. It can be seen that there is a good agreement between predicted values and experimental data as far as S/N ratios for *f*_WC_ and *η* are concerned, with marginal errors varying from 2.30 to 6.78% (see Table 16).

**Table 16:** Selected confirmation tests to improve overlay quality by prioritizing *f*_WC_ and *η*

Confirmation test	*WFS* [m/min]	*TS* [m/min]	*SG* [%]	S/N ratio of *f*_WC_			S/N ratio of *η*		
				Predicted [dBi]	Experimental [dBi]	Error [%]	Predicted [dBi]	Experimental [dBi]	Error [%]
**V1**	2.1	0.3	5% O_2_	30.66	27.07	6.78	38.89	35.59	4.82
**V2**	3.9	0.3	5% O_2_	29.73	28.71	3.43	38.66	37.77	2.30

**Table 17:** Confirmation test results

**ID**	***WFS*** **[m/min]**	***TS*** **[m/min]**	***SG*** **[%]**	***I***_**avg**_** [A]**	***V***_**av*g***_** [V]**	***HI*** **[kJ/mm]**	***AIP*** **[kW]**	***f***_**WC**_ **[%]**	***η*** **[%]**	***DL*** **[%]**	***P* [mm]**	***DR*** **[kg/h]**	***W* [mm]**	***H* [mm]**	***W*/*H***	*θ* [°]
V1	2.1	0.3	5% O_2_	60.15	9.14	0.16	0.79	27.06	71.34	1.01	52.67	1.15	2.91	2.27	1.29	96.33
V2	3.9	0.3	5% O_2_	88.33	9.67	0.26	1.28	27.29	77.46	7.10	184.00	1.87	6.91	2.18	3.17	56.83

The first confirmation test at *WFS* of 2.1 m/min yielded weld bead geometry that is not suitable for weld cladding (albeit with high *f*_WC_ and *η* and with low *DL*). In contrast, the second confirmation test at *WFS* of 3.9 m/min achieved not only the same high level of *f*_WC_ and *η* as the first test but with much-improved weld bead geometry and higher *DR*, achieving much-improved overlay quality. Both tests are designed based on Taguchi analysis, and the outcomes are fully in line with the Taguchi prediction; the desired/predicted improvement of overlay quality is realized through the Taguchi DOE method. In short, the present investigation is a strong testimony of the power and effectiveness of the Taguchi DOE method in process improvement for weld cladding of Ni-WC MMC overlays.

## Conclusion

In this study, Ni-WC MMC overlays were deposited on an A36 steel substrate by employing the CMT process. Taguchi’s design of experiments L16 was used to investigate the effects of CMT processing parameters (*WFS*, *TS*, and *SG*) on *f*_WC_, *η*, *DL*, *DR*, and weld bead geometry. All experiments were successfully deposited with continuous and uniform bead profiles, and no visual defect was detected. The transverse cross-sections exhibit excellent metallurgical bonding to the substrate, an average porosity level of 1.42%, and uniform distribution of retained WC. The main conclusions can be drawn as follows:

1. No degradation seam is observed around the retained WC particles in the deposited samples due to the high dissolution resistance of mono-crystalline WC particles. However, there is inevitably partial dissolution of retained WC particles in the molten pool during deposition, promoting the formation of primary WC and secondary carbides in the matrix;
2. The overall overlay microstructures consist of retained WC fairly uniformly distributed within the matrix of γ-Ni and eutectic Ni/Ni_3_B. The formation of primary WC can be seen in all of the samples. Secondary carbides are also observed, possibly in the form of W_2_C and/or W-rich carbides for samples with low *DL* (i.e., *AIP* around 1.9 kW) and in the form of M_6_C (Ni_2_W_4_C/Fe_3_W_3_C) for those with higher *DL* (i.e., *AIP* > 2 kW), respectively. In general, *f*_WC_ is reduced with increased *DL*, where Fe from the substrate is more easily mixed into the overlay, leading to the excessive dissolution of WC;
3. Dilution is affected by several processing parameters. *HI* and arc impingement have opposing effects on penetration and thus *DL* and should be considered jointly. Higher *DL* (> 15%) is associated with higher *WFS* (5.7–7.5 m/min) and higher *TS* (0.6 m/min), while lower *DL* (1–10%) is observed at lower *WFS* and lower *TS*;
4. *f*_WC_ does not solely depend on *HI* or *AIP*, in contrast to the observations for standard GMAW processes as reported in the open literature. The most influential factor affecting *f*_WC_ is *TS*, followed by *WFS*. High *f*_WC_ (and *η*), together with a high *DR* and improved weld bead appearance, are achievable with the CMT process even at high *HI*;
5. Weld bead characteristics and *DR* are mainly affected by *WFS*. *TS* has a notable effect on weld bead characteristics but has only a marginal effect on *DR*. *HI* and *AIP* are positively correlated with *W*/*H* and *DR*;
6. Improvements in *η* are achieved by using *SG* mixtures with O_2_ additions. This may be due to an increase in the oxidation rate of nickel in the weld pool, improving the arc stability during the droplet formation period, which can eventually lead to an increase in *f*_WC_. In addition, using O_2_ addition results in a decrease in *DL* and *W*/*H*, which remain almost unchanged when using CO_2_. There is no obvious correlation between *DR* and the composition of *SG* mixtures;
7. In general, the processing parameters beneficial to *f*_WC_, *η*, *DL*, and *P* will have a negative effect on *DR* and *W*/*H*. Therefore, it is difficult to find a single set of optimized processing parameters to meet all quality requirements; trade-offs are needed in the engineering practices based on the needs for specific outcomes;
8. Two confirmation tests designed based on Taguchi analysis achieved the expected outcomes; the present investigation clearly demonstrates that the Taguchi DOE method is a powerful tool in process improvement for weld cladding of Ni-WC MMC overlays.

## Data Availability

Not applicable.

## Abbreviations

*η*: Carbide transfer efficiency
*θ*: Contact angle of a weld bead
*ρ*_p_: Density of powders (wrapped within nickel sheath)
*AIP*: Average instantaneous power
*A*_Ni__-sheath_: Nickel sheath area
*A*_P_: Area of weld penetration
*A*_R_: Reinforcement area of a weld bead
*A*_WC_: Total cross-sectional area of WC particles
BSE: Backscattered electrons
CMT: Cold metal transfer
*DL*: Dilution level
DOE: Design of experiments
*DR*: Deposition rate
EDS: Energy-dispersive X-ray spectrometer
*f*_p_: Initial volume fraction of carbide particles in cored feed wire
*f*_WC_: Volume fraction of retained WC
GMAW: Gas metal arc welding
*H*: Height of weld bead
*HI*: Heat input
*I*: Current
*l*_m_: Linear mass of the electrode
Ni-WC MMC: Nickel-based metal matrix composites reinforced with WC
*P*: Penetration depth
*P*_p_: Powder weight percentage
S/N ratio: Signal-to-noise ratio
SEM: Scanning electron microscopy
*SG*: Shielding gas
*TS*: Travel speed
*V*: Voltage
*W*: Width of a weld bead
*W*/*H*: Width to height ratio of a weld bead
WC: Mono-crystalline tungsten carbide
*WFS*: Wire feed speed
XRD: X-ray diffraction
